# Catalytic Reduction of Aqueous Perchlorate at Neutral pH

**DOI:** 10.1021/acscentsci.6c00532

**Published:** 2026-07-08

**Authors:** Jinyu Gao, Shaohua Xie, Jianjun Chen, Qingsong Luo, Juchen Guo, Yadong Yin, Fudong Liu, Jinyong Liu

**Affiliations:** †Department of Chemical and Environmental Engineering and ‡Department of Chemistry, 8790 University of California, Riverside, California 92521, United States

## Abstract

Perchlorate (ClO_4_^–^) is a pervasive and inert pollutant threatening human health. Catalytic reduction of ClO_4_^–^ is imperative for water environment remediation and promising for space exploration. Previous catalysts using oxo Mo^IV^ and Re^V^ species required acidic pH to reduce aqueous ClO_4_^–^. Herein, we developed a ligand-enhanced Ru^0^ nanoparticle catalyst for rapid ClO_4_^–^ reduction by 1 atm H_2_ at pH 7 and 20 °C. Molecular organic nitrogen ligands, particularly *cis*-1,2-diaminocyclohexane (*cis*-DACH), accelerated Ru^0^-catalyzed ClO_4_^–^ reduction by up to 80-fold. Nitrogen-functionalized carbon (NC) support also provided up to 5-fold acceleration. The final [*cis*-DACH]­Ru/NC catalyst demonstrated unprecedented activity and robustness for ClO_4_^–^ reduction at neutral pH. Kinetic analyses and instrumental characterizations found that the nitrogen ligands not only boosted the reactivity of exposed Ru^0^ sites by 63-fold (31-fold by *cis*-DACH and 2-fold by the NC support) but also enhanced ClO_4_^–^ adsorption by 180%. This novel paradigm of tuning nanostructured catalysts by organic ligands will benefit environmental and energy applications for the degradation and utilization of ClO_4_^–^.

## Graphical abstract

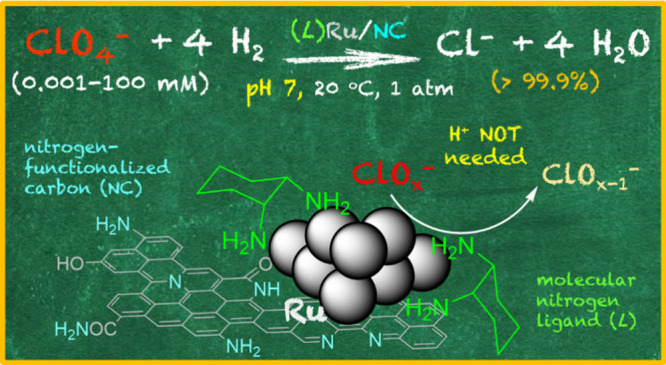

## Introduction

Perchlorate (ClO_4_^–^) is widely distributed in the water environment^1^ due to its natural occurrence^2^ and the manufacture of energetic materials such as rocket fuels, munitions, and pyrotechnics.^3^ Excessive exposure to ClO_4_^–^ can disrupt thyroid hormone production and thus impact the growth, development, metabolism, and mental function of humans.^4^ Recent studies identified new mechanisms of ClO_4_^–^ toxicity in the thyroid, suggesting that its health threat was previously underestimated.^5^ ClO_4_^–^ is also linked to more health issues via nonthyroidal mechanisms.^6^ In the United States, California^7^ and Massachusetts^8^ set the maximum contamination levels for ClO_4_^–^ in drinking water at 6 and 2 μg L^–1^, respectively. In 2023, China began nationwide regulation at 70 μg L^–1^.^9^ In 2026, the United States Environmental Protection Agency resumed efforts toward federal regulation at 20 μg L^–1^.^10^ Besides drinking water, high ClO_4_^–^ contents in turnip, lettuce, spinach, and tea also indicate the need for addressing ClO_4_^–^ contamination in agriculture.^11,12^ Furthermore, the discovery of ClO_4_^–^ on Mars, the moon, and meteorites suggests its widespread presence in the Solar System.^13–16^ Therefore, ClO_4_^–^ reduction technologies are of extensive interest for water purification, food safety, and human extraterrestrial exploration.^17^

In aqueous solution, ClO_4_^–^ is highly inert under various chemical reduction conditions and thus commonly used as an innocent electrolyte in electrochemical systems.^18^ Oxometallates of Mo,^19^ W,^20^ Re,^21^ and Os^22^ catalyzed ClO_4_^–^ reduction by special electron sources (Figure 1a) via oxygen atom transfer (OAT) in the presence of concentrated 1–10 M acids. In contrast, anaerobic bacteria reduce ClO_4_^–^ with hydrogen gas, methane, etc.,^23^ using the Mo cofactor with molybdopterin ligands.^24,25^ The reactivity with ClO_4_^–^ at neutral pH is attributed to the hydrogen bonding between ClO_4_^–^ and an amino acid residue in the enzyme pocket. Similarly, a hydrogen-bonding environment is critical for the high reactivity of biomimetic Fe complexes (moisture-sensitive) with ClO_4_^–^.^26,27^ A biomimetic Mo complex uses Sc^3+^ as a Lewis acid to accelerate the reaction with ClO_4_^–^.^28^ The success of the Fe and Mo complexes has led to the recent advances of using ClO_4_^–^ as a mild oxidizer in organic synthesis for C–H activation and functionalization.^29–31^

**Figure 1:**
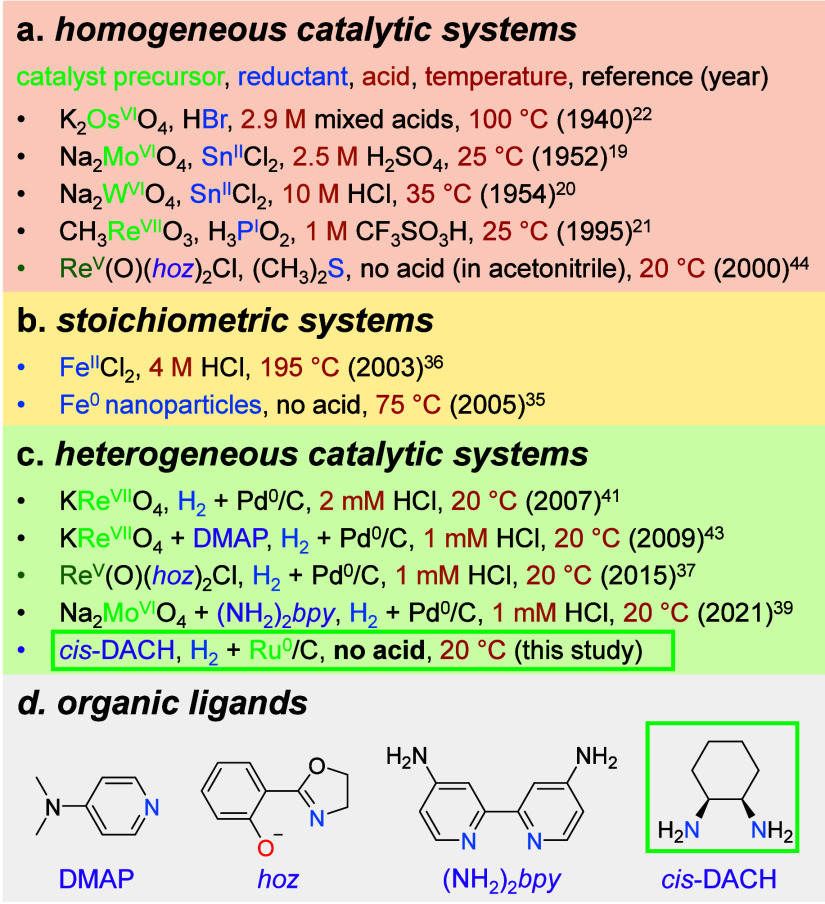
Summary of aqueous perchlorate reduction systems.

In environmental remediation, despite the high activity of perchlorate reductase, microbial reactors typically undergo a long startup phase of several days to months.^32–34^ To meet various needs of ClO_4_^–^ reduction, an abiotic chemical system is highly desirable for both immediate functionality and excellent performance. Earlier environmental remediation efforts used Fe^0^ nanoparticles^35^ and Fe^II^ solution^36^ to reduce ClO_4_^–^, but high temperature and excessive reductants were required (Figure 1b). The enzyme-inspired Re–Pd/C^37,38^ and Mo–Pd/C^39,40^ catalysts (Figure 1c) realized ClO_4_^–^ reduction under mild conditions, such as 1 atm H_2_ as the electron donor, pH 3.0 (1 mM of H^+^), and 20 °C (eq 1):

1 $${{\mathrm{ClO}}_{4}}^{-}+4H_{2}\to {\mathrm{Cl}}^{-}+4H_{2}O$$

These catalysts use Re^V–VII^ or Mo^IV–VI^ redox cycles to enable OAT from ClO_4_^–^.^38,39^ In comparison to catalysts prepared from inorganic KReO_4_ ^41^ and Na_2_MoO_4_ ^42^ precursors, the coordination with select pyridine (N),^43^ oxazoline-phenolate (N–O),^44^ and bipyridine (N–N)^39^ ligands (Figure 1d) accelerated ClO_4_^–^ reduction by 12–5200 fold.^45^ However, the proton-assisted OAT suffers from significant activity loss at pH >4,^37,39,42,46^ preventing the application at neutral pH. Another major challenge is the hydrogenation of aromatic ligands^40,46^ or decomposition of the ligand–metal complex sites,^38,46^ resulting in an irreversible loss of catalyst activity. Interestingly, NO_3_^–^ can deactivate both Re and Mo catalysts developed for ClO_4_^–^ reduction.^40,47^

Herein, we shift the catalyst design rationale to a new paradigm. Among platinum group metal (PGM) nanoparticle catalysts, Ru^0^ has unique reactivity at pH 7.^48^ A bimetallic Ru–Pd/C has exhibited the fastest ClO_3_^–^ reduction to date^49^ but little activity in ClO_4_^–^ reduction. A Ru/CeO_2_ catalyst requires 80 °C to achieve a satisfactory rate of ClO_4_^–^ reduction.^50^ But heating large volumes of water is a major technical obstacle for environmental applications. Although morphology control or support modification might optimize the activity of Ru nanoparticles,^51^ these strategies usually involve laborious procedures. In this study, we modified the properties of Ru^0^ nanoparticles with (i) molecular diamine ligands and (ii) nitrogen functional groups on the support material, enabling the unprecedented ClO_4_^–^ reduction activity at pH 7. We elucidated the contributions of the two nitrogen ligand types and demonstrated the high catalyst robustness under real-world conditions.

## Results

### Substantially Enhanced Perchlorate Reduction by Nitrogen Ligands

Inspired by the in-situ preparation of our earlier Mo catalysts,^39^ we added various nitrogen ligands to the water suspension of a commercial Ru/C catalyst and observed substantially enhanced ClO_4_^–^ reduction at 20 °C. To our surprise, the saturated *cis*-1,2-diaminocyclohexane (*cis*-DACH) provided the highest activity enhancement (Table 1 and Figures S1–S3). Compared to the original Ru/C, the initial turnover frequency (TOF_0_) was accelerated by 80-fold. The activity enhancement by *cis*-DACH was over twice that of its *trans* isomer (Table 1, entry 1 versus 2). The six-mmembered ring backbone also provided a higher activity than other *cis*-1,2-diamino analogs with five- and seven-membered rings (Table 1, entry 1 versus 7 and 8). The *cis*-DACH was also superior to the unsaturated, aromatic, and linear aliphatic diamine ligands (Table 1, entries 5, 6, and 9–11), and to all pyridine-containing ligands (Table 1, entries 12–22). In general, ligands with steric hindrance resulted in lower activity (Table 1, entry 2 versus 3 and 4; entry 9 versus 10 and 11; entry 13 versus 16 and 17), indicating the interaction between Ru and N atoms. The bipyridine (*bpy*) and phenanthroline (*phen*) ligand families also showed higher reaction rates from increased electron-donating character (Table 1, entries 12–15 and 18–20). Therefore, the nitrogen ligands appeared to enhance catalytic performance by altering the electronic properties of the Ru^0^ nanoparticle surface (as characterized in a later section).

**Table 1:**
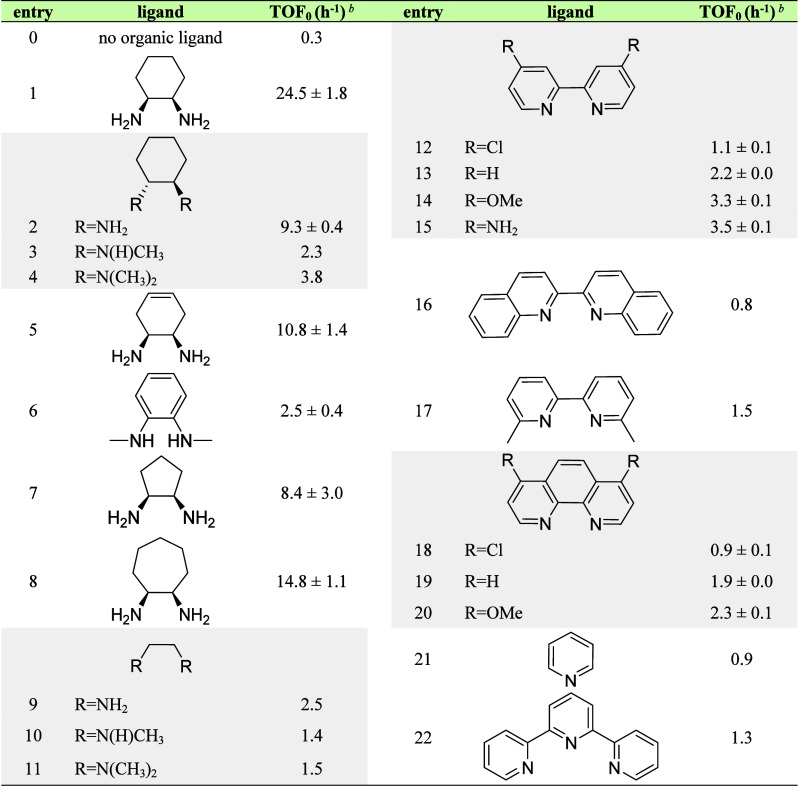
Perchlorate Reduction Activity of Ru/C Enhanced by Various Ligands^a^

a Reaction conditions: 2.0 g L^–1^ of 5 wt % Ru/C (Alfa Aesar No. 44338) with 1 mM ligand added in the water suspension, 1 mM ClO_4_^–^, pH 7.0, 1 atm of H_2_, 20 °C. ^*b*^The calculation assumed that each surface Ru atom reacted with all ClO_*x*_^–^ (*x* = 4, 3, 2, and 1) substrates. Details are described in Text S1 in the Supporting Information.

Having confirmed the positive effect of molecular ligands added from the aqueous solution, we further examined the effect of nitrogen functional groups from the support material. We prepared an amine- and amide-functionalized carbon support (NC) using a “soft nitriding” method with urea at 300 °C.^52^ Then we prepared Ru/C and Ru/NC by incipient wetness impregnation of RuCl_3_ followed by H_2_ reduction at an elevated temperature (detailed procedures in the Supporting Information). Elemental analyses found nitrogen contents of 0.75% and 3.32% in Ru/C and Ru/NC, respectively. Compared with Ru/C, the nitrogen groups in Ru/NC increased the ClO_4_^–^ reduction activity by 460% (Figure 2a). Adding *cis*-DACH to the two catalysts further increased the total nitrogen contents in [*cis*-DACH]­Ru/C and [*cis*-DACH]­Ru/NC to 1.70 and 3.93%, respectively. In the presence of *cis*-DACH, the nitrogen groups in Ru/NC still increased the ClO_4_^–^ reduction activity by 69% (Figure 2a).

**Figure 2:**
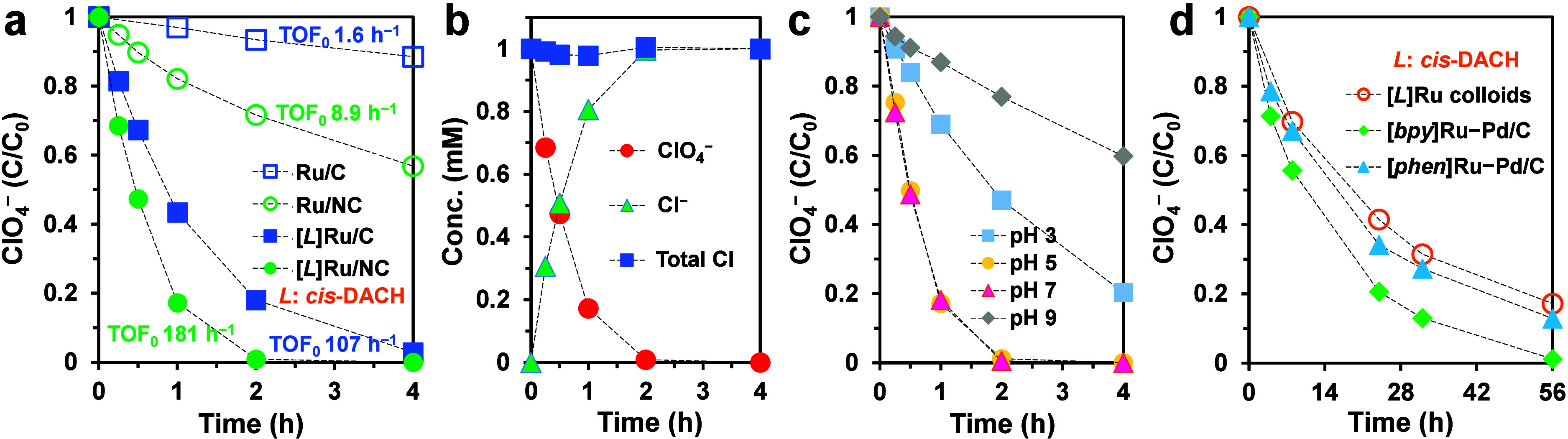
(a) Time profiles of ClO_4_^–^ reduction by Ru catalysts; (b) chlorine balance for ClO_4_^–^ reduction by [*cis*-DACH]­Ru/NC; (c) pH effect on [*cis*-DACH]­Ru/NC. For panels (a)–(c): 2.0 g L^–1^ of lab-prepared catalysts (5 wt % Ru), 2 mM *cis*-DACH added in water. (d) Ligand-enhanced ClO_4_^–^ reduction with Ru colloids (prepared from 1 mM RuCl_3_, 10 mM NaBH_4_, and 10 mM *cis*-DACH) and Ru–Pd/C (2.0 g L^–1^ catalyst; 5 wt % Ru and 5 wt % Pd, 1 mM 2,2′-bipyridine (*bpy*) or 1,10-phenanthroline (*phen*)). For panels (a)–(d): 1 mM ClO_4_^–^, pH 7.0, 1 atm of H_2_, 20 °C.

### Catalyst Performance

At pH 7, the dually enhanced [*cis*-DACH]­Ru/NC catalyst outperformed all reported abiotic catalytic systems in ClO_4_^–^ reduction (Table S1). The reaction rate at 20 °C was similar to that previously reported for Ru/CeO_2_ at 80 °C.^50^ A good mass balance was observed between ClO_4_^–^ and Cl^–^ (Figure 2b), indicating the complete reduction of ClO_4_^–^ to Cl^–^ without accumulating ClO_*x*_^–^ intermediates. The [*cis*-DACH]­Ru/NC and [*cis*-DACH]­Ru/C showed similar responses to solution pH (Figure 2c and Figure S4), with the highest activity at pH 5–7. Because the original Ru/C preferred acidic pH (Figure S5), we attribute the lowered activity at acidic pH to the protonation of −NH_2_, which impeded coordination to Ru^0^ nanoparticles. The reason for the inferior activity at pH 9 for all carbon-supported Ru catalysts remains elusive. However, the previously reported Ru/CeO_2_ catalyst showed similar activity across pH 4–9,^50^ suggesting the promise of combining the organic ligand and novel support materials to maintain activity over a broader pH range. The optimized *cis*-DACH dose was 2 mM (Figure S6), with 59% immobilized on Ru/NC (41% in aqueous solution, determined by total nitrogen analysis). We note that the immobilized ligand (1.18 mM) is more than 10 times the surface Ru atoms in Ru/NC (0.09 mM, calculated using the metal dispersion, Table S2). Thus, the majority of *cis*-DACH was adsorbed on the carbon surface. Lowering the *cis*-DACH dose reduced the dissolved portion (Table S3) at the cost of catalytic activity (Figure S6). After filtration, the redispersion of the collected [*cis*-DACH]­Ru/NC solid in water still released 10% of the initial *cis*-DACH into the aqueous phase. Thus, *cis*-DACH was distributed in an equilibrium between the catalyst surface and water. However, the dissolved *cis*-DACH maintained the same concentration throughout ClO_4_^–^ reduction (Figure S7). In practical application scenarios, the dissolved *cis*-DACH in the treated water could be protonated at low pH and captured by cation exchange resin.^53,54^ The ligand structure could also be further modified with hydrophobic functional groups to substantially reduce the distribution in the aqueous phase.

### Broader Examinations of the Ligand–Nanoparticle Design

We further evaluated the adaptability of the [*cis*-DACH]­Ru/NC design to other metals and systems. However, *cis*-DACH resulted in very limited ClO_4_^–^ reduction for Rh/C and Ir/C and no activity for Pd/C and Pt/C (Figure S8), indicating the critical role of the Ru element. We also confirmed that Ru^0^, rather than Ru^II^ or Ru^III^, is the active site for ClO_4_^–^ reduction. First, mixing RuCl_3_ and *cis*-DACH in water under 1 atm H_2_ provided no activity. The direct reduction of aqueous Ru^III^ by H_2_ under ambient temperature and pressure was sluggish.^49^ Instead, NaBH_4_ effectively reduced Ru^III^ into Ru^0^ colloids (Figure S9)^55^ and the presence of *cis*-DACH enabled ClO_4_^–^ reduction (Figure 2d). Second, adding presynthesized [Ru^II^(*phen*)_3_]^2+^, [Ru^II^(*bpy*)_3_]^2+^, Ru^II^(*bpy*)_2_Cl_2_, and [Ru^II^(NH_3_)_6_]^2+^ complexes (Table S4) into Pd/C under H_2_ did not reduce ClO_4_^–^. In contrast, mixing RuCl_3_, free *phen* or *bpy* ligands, and Pd/C under H_2_ enabled ClO_4_^–^ reduction (Figure 2d). The discrepancy between the two strategies can be attributed to the rapid formation of Ru^0^ nanoparticles from RuCl_3_, which can be substantially accelerated by Pd/C.^49^ The results suggest the active site as ligand-coordinated Ru^0^ nanoparticles rather than single-atom Ru^0^(*ligand*)_*x*_ complexes. However, the performances of [*phen*]­Ru–Pd/C and [*bpy*]­Ru–Pd/C were very similar to that of the initially screened [*phen*]­Ru/C and [*bpy*]­Ru/C (Figures S1a and S1c). Hence, we did not further pursue the incorporation of Pd, which only accelerated the formation of Ru^0^ but not the reduction of ClO_4_^–^.

### Mechanistic Elucidation

After finalizing the catalyst preparation strategy and procedure, we elucidated how *cis*-DACH and the nitrogen groups on NC interacted with Ru and enhanced ClO_4_^–^ reduction. First, the nitrogen ligands did not change the morphology of Ru nanoparticles. Scanning transmission electron microscopy (STEM) characterization showed good dispersion of Ru particles and very similar size distribution (1.6 ± 0.3 nm for Ru/C and 1.5 ± 0.3 nm for Ru/NC; see Figure 3a and b, and Figures S10–S17). The small particle size resulted in no sharp Ru^0^ crystal peaks in X-ray diffraction (XRD) analysis (Figure S18). CO chemisorption revealed very similar Ru dispersion in Ru/C (10.2%) and Ru/NC (12.6%). Therefore, nitrogen groups on the NC support did not result in significantly improved Ru dispersion. Because *cis*-DACH was added to the Ru catalysts at room temperature, as expected, it did not alter the metal morphology (Figure 3c and d). However, the decrease of the apparent dispersion values from 12.6% (Ru/NC) to 3.8% ([*cis*-DACH]­Ru/NC) suggested that *cis*-DACH hindered the coordination of gaseous CO to ∼70% of surface Ru atoms in the dried catalyst. We further used suspended WO_3_ particles in water as a probe of H_2_ activation^56–58^ for the effect of *cis*-DACH coordination. As expected, *cis*-DACH added to Ru/C slowed the reductive conversion of bright yellow WO_3_ to dark blue H_*x*_WO_3_ considerably (Figure 3i versus Figure 3j and Figure S19), indicating that *cis*-DACH also hindered the access of dissolved H_2_ to a significant fraction of surface Ru atoms. Therefore, it is reasonable to postulate that the *cis*-DACH-coordinated Ru atoms cannot bind and reduce aqueous ClO_4_^–^.

**Figure 3:**
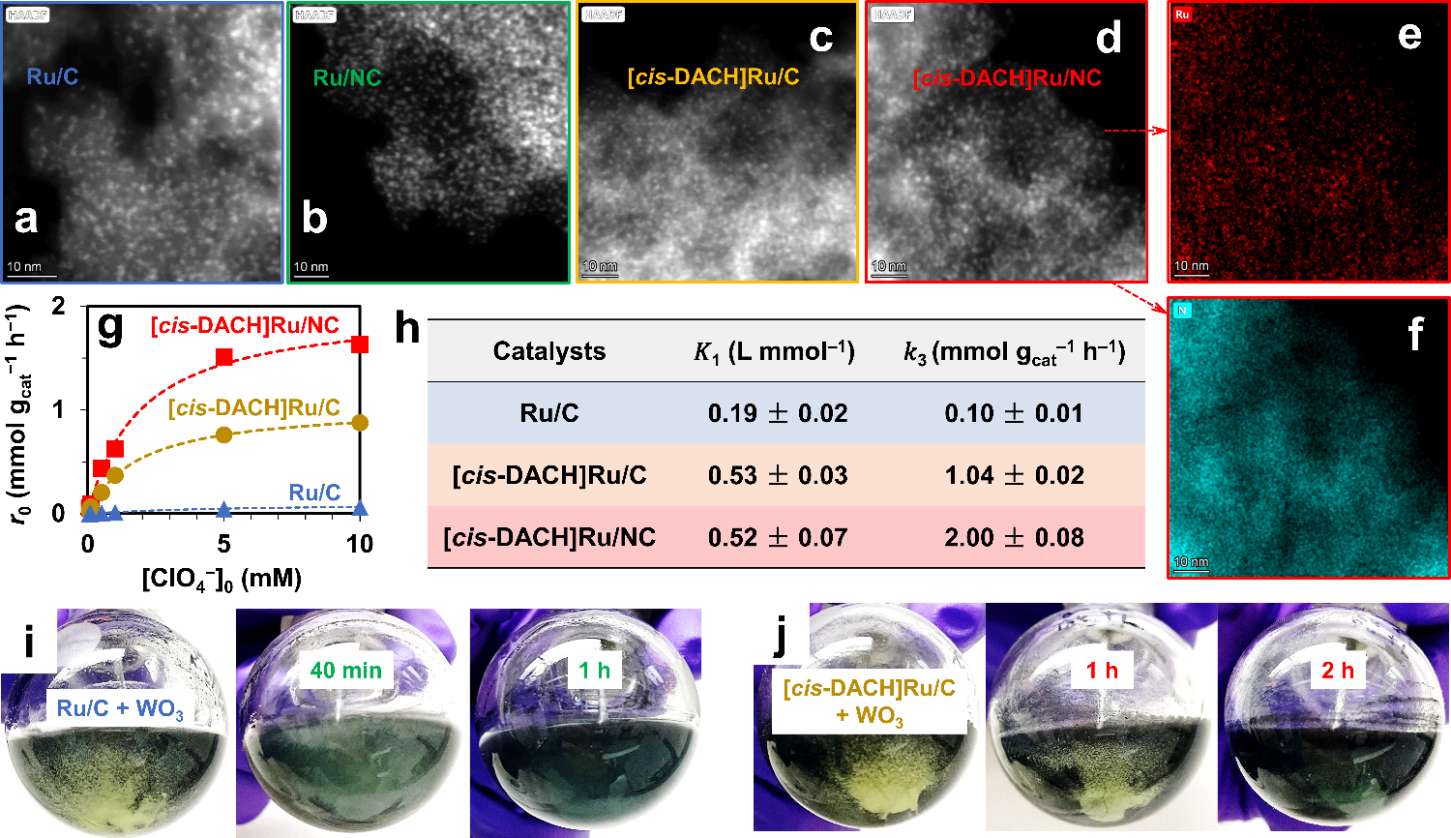
(a–d) HAADF-STEM images of Ru catalysts, and EDX mapping of (e) Ru and (f) N on [*cis*-DACH]­Ru/NC; (g) initial rate of ClO_4_^–^ reduction at various initial ClO_4_^–^ concentrations and the LH model fits; (h) LH model-derived adsorption equilibrium constant *K*_1_ and rate constant *k*_3_ for Ru catalysts at 20 °C; color change of WO_3_ powders (4 g L^–1^) by (i) Ru/C and (j) [*cis*-DACH]­Ru/C under H_2_ sparging at 60 °C. Reaction conditions: 2 g L^–1^ of catalyst (5 wt % Ru), 2 mM *cis*-DACH, pH 7.0, 1 atm of H_2_.

We conducted kinetic analysis with the Langmuir–Hinshelwood (LH) model (eq 2) to differentiate the contributions of *cis*-DACH and the nitrogen groups on the NC:

2 $$r=\frac{K_{1}k_{3}\left[A\right]}{K_{1}\left[A\right]+1}$$

where *r* is the apparent rate of ClO_4_^–^ reduction in the bulk aqueous phase (mmol g_cat_^–1^ h^–1^), K_1_ is the equilibrium constant for ClO_4_^–^ adsorption (L mmol^–1^), *k*_3_ is the rate constant for the reduction of surface-adsorbed ClO_4_^–^ (mmol g_cat_^–1^ h^–1^), and [*A*] is the ClO_4_^–^ concentration in the bulk aqueous phase (mmol L^–1^). Text S2 provides mass transfer analysis and derivation of eq 2. The values of *K*_1_ and *k*_3_ (Figure 3h) were obtained by fitting the LH model with kinetic data from reducing 0.05–10 mM ClO_4_^–^ (Figures 3g and S20). The [*cis*-DACH]­Ru/C catalyst exhibited 180% higher *K*_1_ and 940% higher *k*_3_ than Ru/C. Hence, the addition of *cis*-DACH ligand substantially enhanced both the adsorption and the reduction of ClO_4_^–^. In particular, considering the 70% decrease of exposed Ru sites and that the ClO_4_^–^ reduction could only occur on Ru (see below), the *cis*-DACH coordination on Ru nanoparticles increased the ClO_4_^–^ reduction activity of the remaining (30%) exposed Ru atoms in Ru/C by 31-fold. Further switching the support to NC doubled *k*_3_ but did not yield a higher *K*_1_ for [*cis*-DACH]­Ru/NC. Therefore, nitrogen groups on the NC support could synergistically accelerate the reduction of adsorbed ClO_4_^–^. Although NC materials have been well-known for enhancing the positive surface potential,^59–61^ their role in enhancing ClO_4_^–^ adsorption was overwhelmed by *cis*-DACH. More importantly, as suggested by CO chemisorption data, neither NC support nor *cis*-DACH increased the number of Ru sites. Thus, the increased *k*_3_ could only be attributed to the enhanced activity of Ru nanoparticles. The vastly different activities of Ru, Rh, and Ir catalysts (Figure S8) have suggested that direct interaction between ClO_4_^–^ and Ru is necessary.^48^ In other words, “H spillover” from an H_2_-activating metal particle to a distantly adsorbed substrate on the carbon surface is unlikely to reduce ClO_4_^–^.

X-ray photoelectron spectroscopy (XPS) characterization revealed only one set of Ru 3p spin–orbit doublets for both Ru/C (Figure 4a) and Ru/NC (Figure 4b), with the 3p_3/2_ binding energies (BE) of 462.8 and 462.4 eV, respectively. Both peaks indicate metallic Ru^0^,^62,63^ and the 0.4 eV difference probably reflects changes in the electronic properties of surface Ru atoms due to coordination with nitrogen groups on NC support. The same BE shift was observed between [*cis*-DACH]­Ru/C and [*cis*-DACH]­Ru/NC (Figure 4c versus [Fig fig4]d). In comparison, the addition of *cis*-DACH triggered a minor BE shift of 0.2 eV for both Ru/C (Figure 4c versus [Fig fig4]a) and Ru/NC (Figure 4d versus [Fig fig4]b). Regarding nitrogen speciation in the catalysts, only one N 1s peak (400.1 eV) corresponding to – NH_2_ ^64,65^ was observed in [*cis*-DACH]­Ru/C (Figure 4e). Three N 1s peaks corresponding to −NH_2_ (399.9 eV, major, 49.5% abundance), graphitic N (401.6 eV), and pyridinic N (398.2 eV)^65,66^ were fit for Ru/NC (Figure 4f). [*cis*-DACH]­Ru/NC also contained three species (Figure 4g). The *cis*-DACH addition elevated the −NH_2_ peak abundance from 49.5% to 64.3%.

**Figure 4:**
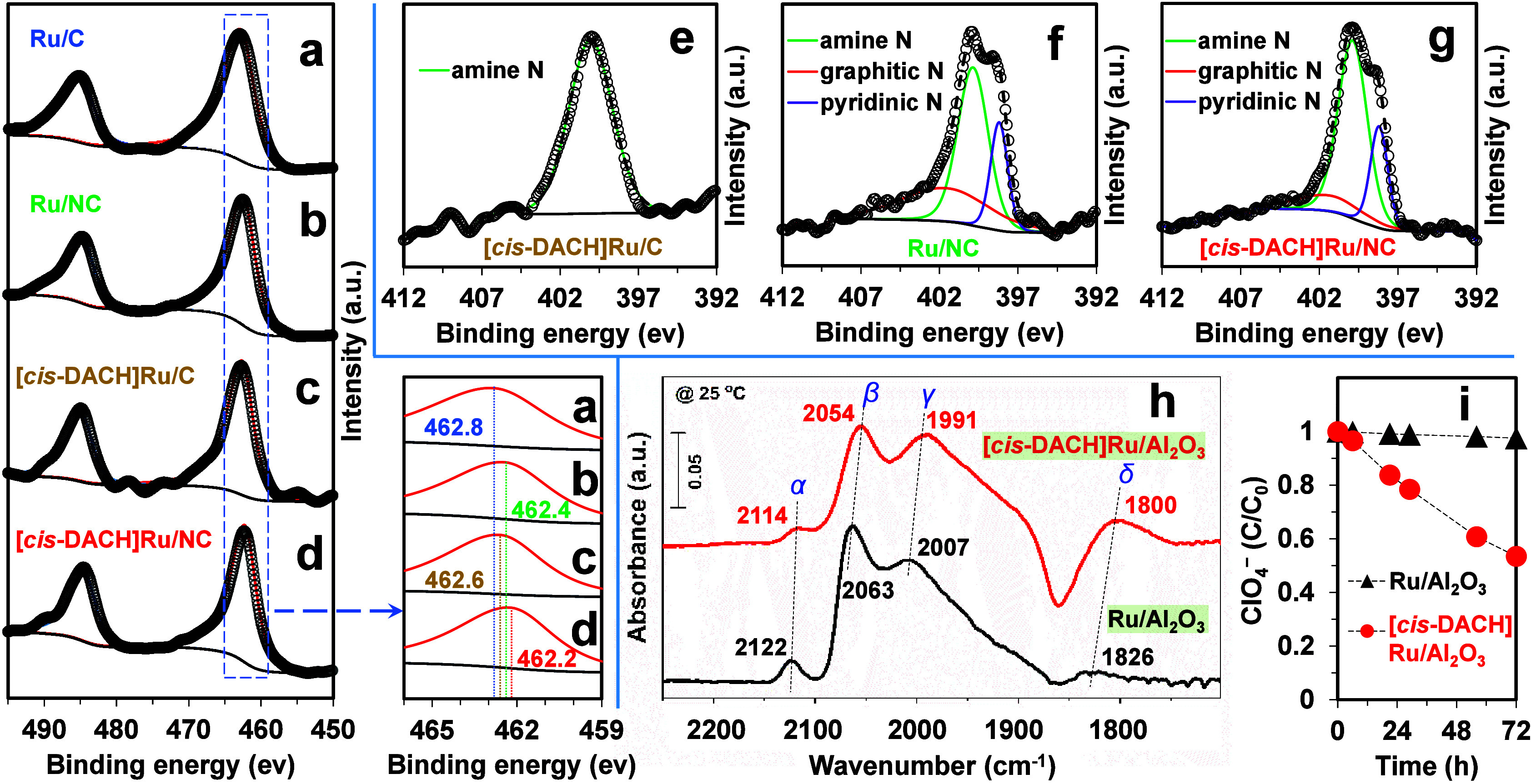
(a–d) Ru 3p XPS spectra (empty dots) and fits (solid lines) of Ru catalysts; (e–g) N 1s XPS spectra (empty dots) and fits (solid lines) of Ru catalysts; (h) DRIFT spectra of Ru catalysts; (i) time profiles of ClO_4_^–^ reduction by Ru/Al_2_O_3_ with and without *cis*-DACH. Reaction conditions: 2 g L^–1^ of 5 wt % Ru/Al_2_O_3_, 2 mM *cis*-DACH, 1 mM ClO_4_^–^, pH 7.0, 1 atm of H_2_, 20 °C.

To further probe the ligand effects on the surface electronic properties of Ru^0^ nanoparticles, we used diffuse-reflectance infrared Fourier transform spectroscopy (DRIFTS) to characterize the CO adsorption at 25 °C (Figure 4h). Since the carbon support can fully absorb IR irradiation, we prepared Ru/Al_2_O_3_ and [*cis*-DACH]­Ru/Al_2_O_3_. On the Ru/Al_2_O_3_ platform, *cis*-DACH also accelerated ClO_4_^–^ reduction by 24-fold (Figure 4i), suggesting that the simple ligand addition method can be applied to multiple catalyst support materials, particularly when nitrogen-doping into the specific solid is difficult. The IR bands corresponding to Ru-multicarbonyl (α, Ru­(CO)_n_ species), on-top adsorbed (β) and bridge-bonded (γ) CO on Ru sites, as well as bridge-bonded CO on interfacial Ru sites (δ)^67^ were observed regardless of the presence of *cis*-DACH. The red shift of all IR bands (8–26 cm^–1^) upon adding *cis*-DACH suggests that the Ru^0^ nanoparticles become more electron-rich. The enhanced back-donation of electrons to the 2π* antibonding orbitals of CO resulted in a lower frequency of molecular stretching. This trend corroborates the XPS-observed shift in the Ru 3p_3/2_ BE upon ligand coordination. Therefore, kinetic analysis of *k*_3_ and material characterization data collectively confirmed that nitrogen ligands donated electrons to Ru^0^ nanoparticles, thereby accelerating ClO_4_^–^ reduction. Notably, this mechanism is distinct from the reported ligand-nanoparticle systems for the hydrogenation of organic substrates,^68^ in which the ligand was added to prevent deeper reactions rather than enhance the reactivity.

### Catalyst Robustness in Real World Situations.

The robustness of the [*cis*-DACH]­Ru/NC catalyst was further demonstrated under various highly challenging water matrices. The catalyst achieved >99.9% reduction of 100 mM ClO_4_^–^ within 50 h (Figure 5a) with the turnover number (TON) of 4350. In the presence of concentrated 0.1 M NaCl, 2 M NaCl, and 1 M Na_2_SO_4_, the activity decreased by 54%, 93%, and 75%, respectively (Figure 5b). After ten spikes of 10 mM ClO_4_^–^ (one spike per day) into the [*cis*-DACH]­Ru/NC suspension, the activity loss observed at day 10 was merely due to the accumulated Cl^–^ from ClO_4_^–^ reduction (Figure 5c). ^1^H NMR analysis found no chemical transformation of *cis*-DACH throughout the catalytic process (Figure S21). The saturated structure of *cis*-DACH is inert to hydrogenation, which was a major challenge for the previously reported Mo–Pd/C catalyst. The latter used 4,4′-diamino-2,2′-bipyridine as the best ligand and was deactivated by 60% under continuous exposure to 1 atm H_2_ for a week.^40^ Moreover, the catalyst prepared from the simple mixing of *cis*-DACH and Ru nanoparticles did not deactivate upon exposure to air. Upon resuming the H_2_ supply, the ClO_4_^–^ reduction activity was fully restored (Figure 5d). The H_2_ atmosphere preserved Ru as metallic nanoparticles, resulting in no detected Ru in the aqueous phase.^49^ Hence, the catalyst showed high promise of treating ClO_4_^–^ in real wastewater, particularly for the straightforward preparation, mild reaction conditions, robustness, longevity, and easy handling.

**Figure 5:**
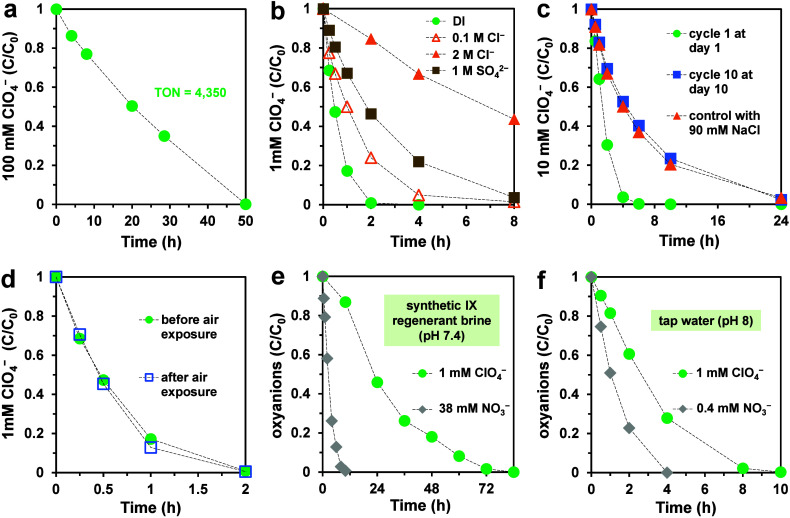
Time profiles of the reduction of (a) 100 mM ClO_4_^–^; (b) 1 mM ClO_4_^–^ in the presence of concentrated Cl^–^ and SO_4_^2–^; (c) 10 mM ClO_4_^–^ by freshly prepared (cycle 1) and reused (cycle 10, with nine previous spikes of 10 mM ClO_4_^–^) catalyst. Each ClO_4_^–^ spike was allowed 24 h to achieve complete reduction into Cl^–^, with ten spikes over a total of 10 days to complete; (d) 1 mM ClO_4_^–^ reduction by [*cis*-DACH]­Ru/NC before and after air exposure for 1 h. The preparation of [*cis*-DACH]­Ru/NC took 1 h under 1 atm H_2_. After air exposure, the catalyst suspension was exposed to 1 atm H_2_ for an additional hour before adding ClO_4_^–^; the reduction of mixed ClO_4_^–^ and NO_3_^–^ in (e) synthetic IX regenerant brine and (f) tap water. Default reaction conditions: 2 g L^–1^ of 5 wt % Ru/NC, 2 mM *cis*-DACH, 1 mM ClO_4_^–^, pH 7, 1 atm of H_2_, 20 °C.

We further tested the performance of [*cis*-DACH]­Ru/NC in real-world scenarios, including ClO_4_^–^ treatment in (i) waste brines resulting from ion-exchange resin regeneration and (ii) drinking water containing ClO_4_^–^ as a disinfection byproduct or from source water. We prepared a synthetic waste brine containing all anions identified in a previous brine treatment study (Table 2).^47^ Notably, due to the low selectivity of early IX resins for ClO_4_^–^, the waste brines produced during resin regeneration often contained significantly higher concentrations of nitrate and sulfate than ClO_4_^–^.^47,70–72^ In this scenario, Re- and Mo-based catalysts require a two-stage treatment for ClO_4_^–^ reduction, as NO_3_^–^ can deactivate them and thus needs to be reduced first by another catalyst (e.g., In–Pd/Al_2_O_3_).^40,47^ In sharp contrast, Ru catalysts can be highly effective in reducing NO_3_^–^,^73^ obviating the need for pretreatment using other catalysts. More than 99% of the 38 mM of NO_3_^–^ was reduced by [*cis*-DACH]­Ru/NC within 10 h (Figure 5e). Although the water matrix substantially slowed the reaction rate (Figure 5e versus 2a), > 99.99% of ClO_4_^–^ was reduced within 84 h. In the tap water that contained 0.4 mM NO_3_^–^ at pH 8 (Table 2), [*cis*-DACH]­Ru/NC reduced >99.99% of NO_3_^–^ and 99.7% of the spiked 1 mM ClO_4_^–^ within 4 and 10 h, respectively (Figure 5f). These positive results further demonstrate the value of [*cis*-DACH]­Ru/NC in practical ClO_4_^–^ treatment, where various case-specific pretreatment processes can be integrated to further enhance the catalyst performance.^45^ Notably, the reaction time can be proportionally shortened by increasing the catalyst loading.^37,39^ Adequate H_2_ supply and catalyst retention at pilot-scale can be achieved by a stirred-tank reactor with microfiltration membrane fibers (Figure S22). Even at a low catalyst loading, this ClO_4_^–^ reduction method does not require intensive energy input, such as heat or UV,^74^ to drive the reaction.

**Table 2:** Composition of Synthetic IX Regenerant Brine and Tap Water

component	concentration in synthetic IX regenerant brine^a^	concentration in tap water^f^
chloride	0.9 M	0.92 mM
perchlorate	1 mM^b^	1 mM^g^
nitrate	38 mM	0.4 mM
sulfate	48 mM	0.66 mM
phosphate	0.22 mM	0.5 μM
fluoride	NA^c^	0.025 mM
alkalinity	7 g/L as CaCO_3_ ^d^	0.17 g/L as CaCO_3_
pH	7.4^e^	8^e^

a Na^+^ was the sole cation introduced alongside the anionic species. Although K^+^, Ca^2+^, and Mg^2+^ are all present in real brines in small quantities, they had negligible effects on reaction kinetics.^46,47^

b The ClO_4_^–^ concentration in the real brine was at 0.02 mM (∼2 mg L^–1^). To ensure accurate ClO_4_^–^ quantitation within the concentrated salt matrix and to compare with the majority of experiments using 1 mM ClO_4_^–^ as the probe, we increased the concentration to 1 mM. The catalyst can effectively reduce >99% of the 0.02 mM ClO_4_^–^ (Figure S20).

c Data was not available in the previous study.^47^

d 0.14 M sodium bicarbonate was added.

e While the reduction of ClO_4_^–^ does not consume H^+^, the reduction of NO_3_^–^ consumes H^+^ and raises the pH. The experiment used a 50 mL double-neck flask with rubber stoppers on both necks. One stopper held two needles for H_2_ inlet and outlet/sampling port, while the other accommodated a pH electrode to monitor pH changes. H_2_SO_4_ (0.1 M) was introduced via the sampling needle when the pH exceeded the initial value (i.e., 7.4 or 8).

f The tap water was sourced from Southern California, with information on the concentrations of other components available in the local water quality report.^69^

g The ClO_4_^–^ concentration in the tap water was below the detection limit of ion chromatography (0.1 μM). We raised it to 1 mM for the accuracy of quantitation and convenient comparison.

## Conclusions

Simply adding an aliphatic *cis*-DACH ligand can substantially enhance the activity of Ru^0^ nanoparticles. This configuration is the first case of rapid ClO_4_^–^ reduction at pH 7 at ambient temperature and pressure. Modifying the carbon support with nitrogen functional groups also enhances the reaction rate. The resulting [*cis*-DACH]­Ru/NC shows excellent performance with a wide range of ClO_4_^–^ concentrations and water matrix conditions. The nitrogen groups in the molecular ligand or the solid support modified the electronic properties of the Ru^0^ nanoparticles, thereby enhancing their reactivity with ClO_4_^–^. The nitrogen groups also enhanced ClO_4_^–^ adsorption to the catalyst surface. The unique activity of Ru, the structure specificity of *cis*-DACH, and the electronic structure of nitrogen-coordinated Ru^0^ nanoparticles warrant further investigation. This study exemplifies a new catalyst design rationale of using organic modifiers to enhance the catalytic performance of metal nanoparticles.

## Materials and Methods

### Chemicals and Materials

RuCl_3_·xH_2_O (99.98%) and NaClO_4_ (≥99%) were used as received from Sigma–Aldrich. Detailed information on support materials and commercial catalysts is described in Table S5. The nitrogen ligands were used as received from Oakwood Chemical, Alfa Aesar, Ark Pharm, Combi–Blocks, Sigma–Aldrich, and TCI. All aqueous solutions were prepared with Milli-Q water (resistivity >18.2 MΩ cm).

### Catalyst Preparation

#### Nitrogen-functionalized carbon (NC)

NC was prepared following a previously reported “soft nitriding” method.^52^ One gram of activated carbon and 1.5 g of urea were mixed and ground in a crucible. The mixture was annealed at 150 °C for 2 h and then at 300 °C for another 2 h. The resulting powder was first washed with ethanol and Milli-Q water to remove the unreacted urea and then dried in a 75 °C oven.

#### Supported Ru catalysts

The Ru^III^Cl_3_ precursor was impregnated into the support material (C, NC, or Al_2_O_3_) by incipient wetness. The resulting wet paste was first dried in an oven at 75 °C for 12 h and then reduced with Ar/H_2_ (v/v = 95/5) at 450 °C for 6 h to yield Ru^0^/C, Ru^0^/NC, and Ru^0^/Al_2_O_3_.^75,76^

#### Ligand-modified Ru catalysts

A 50 mL flask was sequentially loaded with 100 mg of Ru catalysts, 50 mL of DI water, and the desired amount of nitrogen ligand. Since the protonation of nitrogen ligands increased the solution pH, H_2_SO_4_ was used to adjust the pH to 7 (or other designated pH values). After being sealed with a rubber stopper, the flask was sonicated for 1 min to disperse the catalyst particles. The suspension was stirred at 360 rpm and sparged with H_2_ (1 atm) at room temperature (around 20 °C) for 1 h to afford the ligand-modified Ru catalyst suspension (e.g., [*cis*-DACH]­Ru/NC) and allow the reduction of surface oxide on Ru nanoparticles. The H_2_ gas supply was achieved by two 16-gauge stainless steel needles (introduced through the stopper), which served as gas inlet and outlet, respectively.

### Perchlorate reduction and aqueous sample analysis

The reaction was initiated by adding the NaClO_4_ stock solution to the catalyst suspension at 20 °C. Aliquots were collected through the H_2_ outlet needle with a 3 mL plastic syringe and immediately filtered through a 0.22-μm cellulose acetate membrane. ClO_4_^–^ concentration was measured by ion chromatography (Dionex ICS–5000) equipped with a conductivity detector and an IonPac AS16 column. The column was used at 30 °C, with 65 mM KOH eluent at 1 mL min^–1^. The concentration of *cis*-DACH in aqueous samples was determined by total nitrogen analysis of the solution using a Shimadzu TOC-LCSH system equipped with a total nitrogen measurement unit (TNM-L). The control experiment confirmed that nitrogen leaching from the NC material is negligible.

### Catalyst collection and characterization

The freshly prepared catalyst suspension was immediately transferred into an anaerobic glovebag (98% N_2_, 2% H_2_; Coy Laboratories) to avoid artifacts from air oxidation and then filtered under vacuum. The filter paper coated with the catalyst was transferred into a 20 mL scintillation vial and dried in a 110 °C sand bath for 8 h. The dried catalyst powder was stored in the glovebag before further characterization with X-ray photoelectron spectroscopy (XPS), diffuse reflectance FT-IR spectroscopy (DRIFTS), scanning transmission electron microscopy (STEM), and other techniques. Details of catalyst characterizations are described in Text S3.

## Supplementary Material



## Notes

Comparison with other reaction systems; full data of perchlorate reduction kinetics under varying configurations (ligand structure, pH, ligand concentration, metal species, perchlorate concentration), ligand immobilization and transformation (^1^H NMR), and catalyst characterization (metal dispersion, XRD spectra, and HAADF-STEM-EDX images); Ru colloid formation; H_2_ activation tests with the WO_3_ indicator; proposed reactor design; texts for catalyst turnover calculation, mass transfer analysis, and the Langmuir–Hinshelwood kinetics modeling (PDF)

## References

[1] Brandhuber P., Clark S., Morley K. (2009). A review of
perchlorate occurrence
in public drinking water systems. J. Am. Water
Works Assoc..

[2] Dasgupta P. K., Martinelango P. K., Jackson W. A., Anderson T. A., Tian K., Tock R. W., Rajagopalan S. (2005). The origin of naturally occurring
perchlorate: The role of atmospheric processes. Environ. Sci. Technol..

[3] Rehwoldt M. C., Yang Y., Wang H., Holdren S., Zachariah M. R. (2018). Ignition
of nanoscale titanium/potassium perchlorate pyrotechnic powder: Reaction
mechanism study. J. Phys. Chem. C.

[4] Greer M. A., Goodman G., Pleus R. C., Greer S. E. (2002). Health effects assessment
for environmental perchlorate contamination: The dose response for
inhibition of thyroidal radioiodine uptake in humans. Environ. Health Perspect..

[5] Llorente-Esteban A., Manville R. W., Reyna-Neyra A., Abbott G. W., Amzel L. M., Carrasco N. (2020). Allosteric regulation
of mammalian Na^+^/I^–^ symporter activity
by perchlorate. Nat. Struct. Mol. Biol..

[6] Niziński P., Błażewicz A., Kończyk J., Michalski R. (2021). Perchlorate – properties, toxicity and human
health effects: An updated review. Rev. Environ.
Health.

[7] California State Water Resources Control Board . Perchlorate in Drinking Water. Available via the Internet at: https://www.waterboards.ca.gov/drinking_water/certlic/drinkingwater/Perchlorate.html (accessed May 11, 2026).

[8] Massachusetts Department of Environmental Protection. Perchlorate Background Information and Standards. Available via the Internet at: https://www.mass.gov/lists/perchlorate-background-information-and-standards#perchlorate---final-standards- (accessed May 11, 2026).

[9] United States Department of Agriculture, Foreign Agricultural Service. China: National Standard for Drinking Water Quality Released (CH2023–0094). Available via the Internet at: https://www.fas.usda.gov/data/china-national-standard-drinking-water-quality-released (accessed May 11, 2026).

[10] United States Environmental Protection Agency. Perchlorate in Drinking Water. Available via the Internet at: https://www.epa.gov/sdwa/perchlorate-drinking-water#proposed-perchlorate (accessed May 11, 2026).

[11] Arcella D., Binaglia M., Vernazza F., European
Food Safety Authority (2017). Dietary exposure assessment
to perchlorate in the European population. EFSA
J..

[12] EFSA
Panel on Contaminants in the Food Chain (CONTAM) (2014). Scientific opinion on the risks to
public health related to the presence of perchlorate in food, in particular
fruits and vegetables. EFSA J..

[13] Hecht M. H., Kounaves S. P., Quinn R. C., West S. J., Young S. M. M., Ming D. W., Catling D. C., Clark B. C., Boynton W. V., Hoffman J., DeFlores L. P., Gospodinova K., Kapit J., Smith P. H. (2009). Detection of perchlorate
and the
soluble chemistry of martian soil at the Phoenix lander site. Science.

[14] Kim Y. S., Wo K. P., Maity S., Atreya S. K., Kaiser R. I. (2013). Radiation-induced
formation of chlorine oxides and their potential role in the origin
of martian perchlorates. J. Am. Chem. Soc..

[15] Jackson W.
A., Davila A. F., Sears D. W. G., Coates J. D., McKay C. P., Brundrett M., Estrada N., Böhlke J. K. (2015). Widespread
occurrence of (per)­chlorate in the Solar System. Earth Planet. Sci. Lett..

[16] Kounaves S. P., Carrier B. L., O’Neil G. D., Stroble S. T., Claire M. W. (2014). Evidence
of martian perchlorate, chlorate, and nitrate in Mars meteorite EETA79001:
Implications for oxidants and organics. Icarus.

[17] Davila A. F., Willson D., Coates J. D., McKay C. P. (2013). Perchlorate on Mars:
A chemical hazard and a resource for humans. Int. J. Astrobiol..

[18] Chen S., Lan R., Humphreys J., Tao S. (2020). Perchlorate based “oversaturated
gel electrolyte” for an aqueous rechargeable hybrid Zn–Li
battery. ACS Appl. Energy Mater..

[19] Haight G., Sager W. (1952). Evidence for
preferential one-step
divalent changes in the molybdate-catalyzed reduction of perchlorate
by stannous ion in sulfuric acid solution. J.
Am. Chem. Soc..

[20] Haight G. (1954). Mechanism of the
tungstate catalyzed reduction of perchlorate
by stannous chloride. J. Am. Chem. Soc..

[21] Abu-Omar M. M., Espenson J. H. (1995). Facile abstraction of successive oxygen atoms from
perchlorate ions by methylrhenium dioxide. Inorg.
Chem..

[22] Crowell W. R., Yost D. M., Roberts J. D. (1940). The catalytic effect of osmium compounds
on the reduction of perchloric acid by hydrobromic acid. J. Am. Chem. Soc..

[23] Coates J. D., Achenbach L. A. (2004). Microbial
perchlorate reduction: Rocket-fuelled metabolism. Nat. Rev. Microbiol..

[24] Schwarz G., Mendel R. R., Ribbe M. W. (2009). Molybdenum cofactors, enzymes and
pathways. Nature.

[25] Youngblut M. D., Tsai C.-L., Clark I. C., Carlson H. K., Maglaqui A. P., Gau-Pan P. S., Redford S. A., Wong A., Tainer J. A., Coates J. D. (2016). Perchlorate reductase
is distinguished by active site
aromatic gate residues. J. Biol. Chem..

[26] Ford C. L., Park Y. J., Matson E. M., Gordon Z., Fout A. R. (2016). A bioinspired
iron catalyst for nitrate and perchlorate reduction. Science.

[27] Drummond M. J., Miller T. J., Ford C. L., Fout A. R. (2020). Catalytic perchlorate
reduction using iron: Mechanistic insights and improved catalyst turnover. ACS Catal..

[28] Chen S. L., Elrod L. T., Marriott S., Zhao Z., Liu B. Z., Robinson J. R., Kim E. (2025). Nitrate and
perchlorate reduction
by a dinuclear Mo­(V) complex. Inorg. Chem..

[29] Sarkar W., LaDuca A., Wilson J. R., Szymczak N. K. (2024). Iron-catalyzed C–H
oxygenation using perchlorate enabled by secondary sphere hydrogen
bonds. J. Am. Chem. Soc..

[30] Sarkar W., Szymczak N. K. (2025). Expanding perchlorate
use for C–H oxidative
transformations: A tandem photo- and iron-catalytic strategy. Organometallics.

[31] Xu X., Hua K., Zhang M.-T. (2025). Two-phase
perchlorate activation enabled by a dinuclear
Fe-NHC (N-heterocyclic carbene) complex. Angew.
Chem., Int. Ed..

[32] Luo Y.-H., Chen R., Wen L.-L., Meng F., Zhang Y., Lai C.-Y., Rittmann B. E., Zhao H.-P., Zheng P. (2015). Complete perchlorate
reduction using methane as the sole electron donor and carbon source. Environ. Sci. Technol..

[33] Wu H., Zeng X., Liu H., Liu H., Xie B. (2026). Microbial
purification of perchlorate in a simulated martian water to ensure
its plant cultivation for martian BLSS. Acta
Astronaut..

[34] Zhang L.-D., Lai C.-Y., Oren Y., Gilron J., Ronen Z., Zhao H.-P. (2026). Anion-exchange membrane coupled with methane-fed biofilm
enables efficient co-removal of perchlorate and nitrate. Water Res..

[35] Cao J., Elliott D., Zhang W.-x. (2005). Perchlorate reduction by nanoscale
iron particles. J. Nanopart. Res..

[36] Gu B., Dong W., Brown G. M., Cole D. R. (2003). Complete degradation
of perchlorate in ferric chloride and hydrochloric acid under controlled
temperature and pressure. Environ. Sci. Technol..

[37] Liu J., Choe J. K., Wang Y., Shapley J. R., Werth C. J., Strathmann T. J. (2015). Bioinspired
complex-nanoparticle hybrid catalyst system
for aqueous perchlorate reduction: Rhenium speciation and its influence
on catalyst activity. ACS Catal..

[38] Ren C., Liu J. (2021). Bioinspired catalytic
reduction of aqueous perchlorate by one single-metal
site with high stability against oxidative deactivation. ACS Catal..

[39] Ren C., Yang P., Sun J., Bi E. Y., Gao J., Palmer J., Zhu M., Wu Y., Liu J. (2021). A bioinspired
molybdenum catalyst for aqueous perchlorate reduction. J. Am. Chem. Soc..

[40] Ren C., Bi E. Y., Gao J., Liu J. (2022). Molybdenum-catalyzed
perchlorate reduction: Robustness, challenges, and solutions. ACS ES&T Eng..

[41] Hurley K. D., Shapley J. R. (2007). Efficient heterogeneous
catalytic reduction of perchlorate
in water. Environ. Sci. Technol..

[42] Ren C., Yang P., Gao J., Huo X., Min X., Bi E. Y., Liu Y., Wang Y., Zhu M., Liu J. (2020). Catalytic reduction of aqueous chlorate with MoO_x_ immobilized
on Pd/C. ACS Catal..

[43] Hurley K. D., Zhang Y., Shapley J. R. (2009). Ligand-enhanced
reduction of perchlorate
in water with heterogeneous Re–Pd/C catalysts. J. Am. Chem. Soc..

[44] Abu-Omar M. M., McPherson L. D., Arias J., Béreau V. M. (2000). Clean and
efficient catalytic reduction of perchlorate. Angew. Chem., Int. Ed..

[45] Liu J., Gao J. (2023). Catalytic reduction
of water pollutants: Knowledge gaps, lessons
learned, and new opportunities. Front. Environ.
Sci. Eng..

[46] Liu J., Chen X., Wang Y., Strathmann T. J., Werth C. J. (2015). Mechanism and mitigation
of the decomposition of an
oxorhenium complex-based heterogeneous catalyst for perchlorate reduction
in water. Environ. Sci. Technol..

[47] Liu J., Choe J. K., Sasnow Z., Werth C. J., Strathmann T. J. (2013). Application
of a Re–Pd bimetallic catalyst for treatment of perchlorate
in waste ion-exchange regenerant brine. Water
Res..

[48] Chen X., Huo X., Liu J., Wang Y., Werth C. J., Strathmann T. J. (2017). Exploring
beyond palladium: Catalytic reduction of aqueous oxyanion pollutants
with alternative platinum group metals and new mechanistic implications. Chem. Eng. J..

[49] Gao J., Xie S., Liu F., Liu J. (2023). Preparation and synergy
of supported
Ru^0^ and Pd^0^ for rapid chlorate reduction at
pH 7. Environ. Sci. Technol..

[50] Yoshida D., Liu J., Huang K., Otomo R., Kamiya Y. (2023). Reduction of perchlorate
in neutral water over a ceria-supported ruthenium catalyst towards
the purification of contaminated water. Appl.
Catal. A: Gen..

[51] Axet M. R., Philippot K. (2020). Catalysis with colloidal ruthenium
nanoparticles. Chem. Rev..

[52] Liu B., Yao H., Song W., Jin L., Mosa I. M., Rusling J. F., Suib S. L., He J. (2016). Ligand-free
noble metal nanocluster
catalysts on carbon supports via “soft” nitriding. J. Am. Chem. Soc..

[53] Kamionka, M. ; Conradie, A. Materials and methods for the selective recovery of multivalent products. U.S. Patent No. 10,576,467B2 2020.

[54] Rhee, I. H. ; Park, B. G. ; Jung, H. J. Method for recovering amine from amine-containing waste water. U.S. Patent No. 8,545,704B2 2013.

[55] Yu W., Liu M., Liu H., Ma X., Liu Z. (1998). Preparation,
characterization,
and catalytic properties of polymer-stabilized ruthenium colloids. J. Colloid Interface Sci..

[56] Wang C., Guan E., Wang L., Chu X., Wu Z., Zhang J., Yang Z., Jiang Y., Zhang L., Meng X., Gates B. C., Xiao F.-S. (2019). Product
selectivity
controlled by nanoporous environments in zeolite crystals enveloping
rhodium nanoparticle catalysts for CO_2_ hydrogenation. J. Am. Chem. Soc..

[57] Wang S., Hu R., Ren J., Lv Y., Song L., Zhao H., Jiang X., Gao D., Chen G. (2024). Surface hydrophobization
of zeolite enables mass transfer matching in gas-liquid-solid three-phase
hydrogenation under ambient pressure. Nat. Commun..

[58] Luo Q., Wang H., Lv Y., Fan J., Wu H., Fang W., Liu L., Liu P., Wang L., Xiao F.-S. (2025). Hydrophobic poly­(divinylbenzene)
polymer-supported
ruthenium catalysts for efficient hydrogenation of pyridines in water. Angew. Chem., Int. Ed..

[59] Yang M., Lian R., Zhang X., Wang C., Cheng J., Wang X. (2022). Photocatalytic cyclization
of nitrogen-centered radicals with carbon
nitride through promoting substrate/catalyst interaction. Nat. Commun..

[60] Zhang Z., Cheng J., Luo Y., Shi W., Wang W., Zhang B., Zhang R., Bao X., Guo Y., Cui F. (2018). Pt nanoparticles supported on amino-functionalized
SBA-15 for enhanced
aqueous bromate catalytic reduction. Catal.
Commun..

[61] Ding D., Yang S., Qian X., Chen L., Cai T. (2020). Nitrogen-doping
positively whilst sulfur-doping negatively affect the catalytic activity
of biochar for the degradation of organic contaminant. Appl. Catal. B: Environ..

[62] Bock C., Paquet C., Couillard M., Botton G. A., MacDougall B. R. (2004). Size-selected
synthesis of PtRu nano-catalysts: Reaction and size control mechanism. J. Am. Chem. Soc..

[63] Antonetti C., Oubenali M., Raspolli
Galletti A.
M., Serp P., Vannucci G. (2012). Novel microwave synthesis of ruthenium nanoparticles
supported on carbon nanotubes active in the selective hydrogenation
of p-chloronitrobenzene to p-chloroaniline. Appl. Catal. A: Gen..

[64] Kelemen S. R., Afeworki M., Gorbaty M. L., Kwiatek P. J., Solum M. S., Hu J. Z., Pugmire R. J. (2002). XPS and ^15^N NMR study
of nitrogen forms in carbonaceous solids. Energy
Fuels.

[65] Pietrzak R. (2009). XPS study
and physico-chemical properties of nitrogen-enriched microporous activated
carbon from high volatile bituminous coal. Fuel.

[66] Wei D., Liu Y., Wang Y., Zhang H., Huang L., Yu G. (2009). Synthesis
of N-doped graphene by chemical vapor deposition and its electrical
properties. Nano Lett..

[67] Chen S., Abdel-Mageed A. M., Dyballa M., Parlinska-Wojtan M., Bansmann J., Pollastri S., Olivi L., Aquilanti G., Behm R. J. (2020). Raising the CO_x_ methanation activity of
a Ru/γ-Al_2_O_3_ catalyst by activated modification
of metal–support interactions. Angew.
Chem., Int. Ed..

[68] Liu K., Qin R., Zheng N. (2021). Insights into
the interfacial effects in heterogeneous
metal nanocatalysts toward selective hydrogenation. J. Am. Chem. Soc..

[69] Riverside Public Utilities. Riverside public utilities 2024 water quality report. Available via the Internet at: https://www.riversideca.gov/utilities/sites/riversideca.gov.utilities/files/Water%20Quality%20Report_2024.pdf (accessed May 11, 2026).

[70] Batista, J. R. ; McGarvey, F. X. ; Vieira, A. R. , The removal of perchlorate from waters using ion-exchange resins. In Perchlorate in the Environment; Urbansky, E. T. , Ed.; Springer US: Boston, MA, 2000; pp 135–145.

[71] Gingras T. M., Batista J. R. (2002). Biological reduction of perchlorate
in ion exchange
regenerant solutions containing high salinity and ammonium levels. J. Environ. Monit..

[72] Lehman S. G., Badruzzaman M., Adham S., Roberts D. J., Clifford D. A. (2008). Perchlorate
and nitrate treatment by ion exchange integrated with biological brine
treatment. Water Res..

[73] Huo X., Van Hoomissen D. J., Liu J., Vyas S., Strathmann T. J. (2017). Hydrogenation
of aqueous nitrate and nitrite with ruthenium catalysts. Appl. Catal. B: Environ..

[74] Zhang Z., Yin E., Fu Q., Zhang S., Rao D., Gao J., Liu J. (2026). Perchlorate
reduction with integrated photochemical and catalytic
processes. Environ. Sci. Technol. Lett..

[75] Lin B., Wei K., Ni J., Lin J. (2013). KOH activation of thermally modified
carbon as a support of Ru catalysts for ammonia synthesis. ChemCatChem..

[76] Lin B., Heng L., Fang B., Yin H., Ni J., Wang X., Lin J., Jiang L. (2019). Ammonia synthesis
activity
of alumina-supported ruthenium catalyst enhanced by alumina phase
transformation. ACS Catal..

