# Supplementary material for: Catalytic Reduction of Aqueous Perchlorate at Neutral pH
Source: ACS Cent Sci. 2026 Jul 8;12(7):1038–47. doi: 10.1021/acscentsci.6c00532 (PMC13397286; doi:10.1021/acscentsci.6c00532)
Supplement: Supplementary file 1 [file oc6c00532_si_001.pdf]

Supporting Information

Catalytic Reduction of Aqueous Perchlorate at Neutral pH

Jinyu Gao,[1,*] Shaohua Xie,[1] Jianjun Chen,[1] Qingsong Luo,[1] Juchen Guo,[1] Yadong Yin,[2] Fudong Liu,[1] and Jinyong Liu[1,*]

[1]Department of Chemical and Environmental Engineering, [2]Department of Chemistry, University of California, Riverside, California 92521, United States

Corresponding Author
*(J.L.) E-mail: jinyongl@ucr.edu; jinyong.liu101@gmail.com
*(J.G.) E-mail: jgao034@ucr.edu; jinyu.gao001@gmail.com

Table of Contents

Tables S1−S5 Referred to in the Main Text **S2**
Figures S1−S22 Referred to in the Main Text **S5**
Text S1−S3 Referred in the Main Text **S24**
References **S28**

Tables S1−S5 Referred to in the Main Text

Table S1. Detailed Performance Data for ClO_4^- Reduction by Various Reductive Systems.

entry	catalyst (loading)/(reductant)	pH	temp. (°C)	$[ClO_4^-]_0$ (mM)	TON	TOF (h^{-1})	apparent rate constant [a] ($L\ h^{-1}\ g_{metal}^{-1}$)	space-time yield [b] ($g_{ClO4-}\ L^{-1}\ h^{-1}$)	ref
	**heterogeneous catalysts[c] used at circumneutral pH**								
1	**[*cis*-DACH|Ru/NC**<br>(2 g L^{-1})/(1 atm of H_2)	7.0	20	1	144	181	20.3	0.20	this work
2	**[*cis*-DACH|Ru/C**<br>(2 g L^{-1})/(1 atm of H_2)	7.0	20	1	145	107	11.3	0.11	this work
3	Ru/CeO_2<br>(1 g L^{-1})/(10 mL min^{-1} H_2)	7.0	25	1	N.A.[d]	N.A.[d]	2	0.006	1
4	Ru/CeO_2<br>(1 g L^{-1})/(10 mL min^{-1} H_2)	7.0	80	1	30.6	15.3	23	0.069	1
5	Re(O)(*hoz*)$_2$−Pd/C<br>(0.5 g L^{-1})/(1 atm of H_2)	6.0	25	1	N.A.[d]	N.A.[d]	0.3	0.002	2
	**heterogeneous catalysts[c] used at acidic pH**								
6	**[*cis*-DACH|Ru/NC**<br>(2 g L^{-1})/(1 atm of H_2)	3.0	20	1	144	57	5.4	0.054	this work
7	**[*cis*-DACH|Ru/C**<br>(2 g L^{-1})/(1 atm of H_2)	3.0	20	1	145	53	5.2	0.052	this work
8	Ru/CeO_2<br>(1 g L^{-1})/(10 mL min^{-1} H_2)	4.0	80	1	30.6	18.1	30.3	0.090	1
9	[$(NH_2)_2$*bpy*]MoO_x−Pd/C<br>(0.2 g L^{-1})/(1 atm of H_2)	3.0	20	1	38.5	106	140	0.28	3
10	ReO_x−Pd/C<br>(2 g L^{-1})/(1 atm of H_2)	2.7	23	2	13	4.2	1.5	0.060	4
11	[Re(O)$_2$(Me$_2$N*py*)$_4$]−PdC<br>(1 g L^{-1})/(1 atm of H_2)	2.7	20	2	40	31	9.2	0.18	5
12	Re(O)(*hoz*)$_2$−Pd/C<br>(0.5 g L^{-1})/(1 atm of H_2)	3.0	25	1	30	72	50	0.25	2
	**homogeneous catalysts[e]**								
13	K_2OsBr_6<br>(3.8 mM)/(1.2 M HBr)	2.9 M acids	100	1.0 M	6.3 (96 h)	0.47	N.A.	0.18	6
14	Na_2WO_4<br>(1 mM)/(0.09 M $SnCl_2$)	10 M HCl	35	23.3	N.A.	0.20	8.6<br>L mol^{-1} h^{-1}	0.02[f]	7
15	CH_3ReO_3<br>/(H_3PO_2)[g]	1 M CF_3SO_3H	25	N.A.	N.A.	26.3[h]	7.3<br>L mol^{-1} s^{-1}	2.6[f,h]	8
16	Re(O)(*hoz*)$_2$Cl<br>(3 mM)/(0.5 M sulfide)	no acid[i]	20	92	31	7.8	100 %<br>after 4 h	2.3	9
	**stoichiometric**								
18	Fe^0 nanoparticles<br>(10 g L^{-1})	no acid	75	~2	-	-	1.52<br>mg g_{Fe}^{-1} h^{-1}	0.015	10
17	$FeCl_2$<br>(16 equiv)	4 M HCl	195	87-91	-	-	5.2 h^{-1}	45.3	11

[a]Normalized to the mass of metal (Ru, Mo+Pd total, Re+Pd total) for cross-comparison of catalysts with variable metal contents.
[b]Derived from the rate constant and $[ClO_4^-]_0$ under the listed conditions. Note that this parameter varies with catalyst loading and $[ClO_4^-]_0$. For example, a 10x catalyst loading in the water suspension will result in a 10x space-time yield for perchlorate reduction (see Figure S6c versus S6d in our earlier study on a Mo−Pd/C catalyst[3]).
[c]All catalysts had a nominal metal loading of 5 wt% for Ru, Mo, and Pd, except Ru/CeO_2, which contained 3 wt% Ru.
[d]The ClO_4^- concentration−time profiles at the particular pH or temperature were not available in the references.
[e]The reports on homogeneous systems did not contain adequate information to derive all the performance data in this table. The perchlorate reduction kinetics were not pseudo-first-order as observed in the recent heterogeneous systems. A Na_2MoO_4+$SnCl_2$ system[12] (Figure 1a) is not included here because the performance data could not be readily extracted.
[f]The perchlorate reduction rates were calculated as $r_{ClO4-} = k[\text{catalyst}][ClO_4^-]$, where k is the rate constant listed in the table.
[g]The catalyst and reductant loadings were not explicitly reported.
[h]The $[ClO_4^-]_0$ value was not reported, so we used 1.0 mM to calculate and directly compare with heterogeneous systems.
[i]This system used a CH_3CN/H_2O (95:5, v/v) mixed solvent and did not add acid.

Table S2. Metal Content and Metal Dispersion[a] for Various Catalysts

Catalyst	Metal content	Metal dispersion
Ru/C[b]	3.67 %	10.2 %
Ru/NC[b]	3.68 %	12.6 %
Commercial Ru/C	3.67 %	11.6 %

[a]Measured from CO chemisorption. The stoichiometry for Ru:CO is 12:7.[13]
[b]The catalysts were prepared by a conventional method involving incipient wetness impregnation and reduction with heated H_2 (see Method section for details).

Table S3. Percentage of *cis*-DACH Ligand Immobilized on Ru/NC and Ru/C.[a]

cis-DACH (mM)	Immobilized on Ru/NC (%)	Immobilized on Ru/C (%)
0.5	75.4	77.9
1	73.9	69.5
2	59.4	55.1

[a]Concentration of *cis*-DACH in aqueous samples was measured based on the total nitrogen in the solution using a Shimadzu TOC-LCSH system equipped with a total nitrogen measurement unit (TNM-L).

The immobilized percentage = $\frac{(\text{added } cis\text{−DACH}) - (\text{aqueous } cis\text{−DACH})}{\text{added } cis\text{−DACH}}$

Table S4. Commercial Ru^{II} Complexes Tested for ClO_4^- Reduction with Pd/C and H_2.[a]

CAS #	Chemical Name	Formula	Purity
207802-45-7	Tris(1,10-phenanthroline)dichlororuthenium(II) hydrate	$[Ru(phen)_3]Cl_2 \cdot xH_2O$	98%
15305-72-3	Hexaammineruthenium(II) chloride	$[Ru(NH_3)_6]Cl_2$	99.9%
98014-14-3	*cis*-Bis(2,2'-bipyridine)dichlororuthenium(II) hydrate	*cis*-$Ru(bpy)_2Cl_2.xH_2O$	97%
50525-27-4	Tris(2,2'-bipyridyl)dichlororuthenium(II) hexahydrate	$[Ru(bpy)_3]Cl_2 \cdot 6H_2O$	99.95%

[a]Reaction conditions: 2 g L^{-1} of 5 wt % Pd/C catalyst with 1 mM Ru^{II} complexes (equivalent to ~5 wt % as Ru if immobilized), 1 mM ClO_4^-, pH 7, 1 atm of H_2, 20 °C. None of these Ru^{II} complexes showed activity in ClO_4^- reduction.

Table S5. Support Materials for Lab-Prepared Catalysts and Commercial PGM Catalysts.

material	brief description	vendor and catalog #
carbon powder	Norit GSX; steam activated and acid washed; surface area of 1300 $m^2\ g^{-1}$ provided by the vendor; used as received.	Alfa Aesar L11860
aluminum oxide	1/8" pellets for catalyst support; ground into powders before use.	Alfa Aesar 43855
5 wt% Ru/C	wet support (dried at 70°C under air before use).	Alfa Aesar 44338
5 wt% Pd/C	Degussa type E101 NO/W; wet support (dried at 70°C under air before use).	Sigma−Aldrich 330116
5 wt% Rh/C	wet support (dried at 70°C under air before use).	Alfa Aesar 11761
5 wt% Pt/C	wet support (dried at 70°C under air before use).	Alfa Aesar 46306
1 wt% Ir/C	wet support (dried at 70°C under air before use).	Alfa Aesar 38330

Figures S1−S22 Referred to in the Main Text

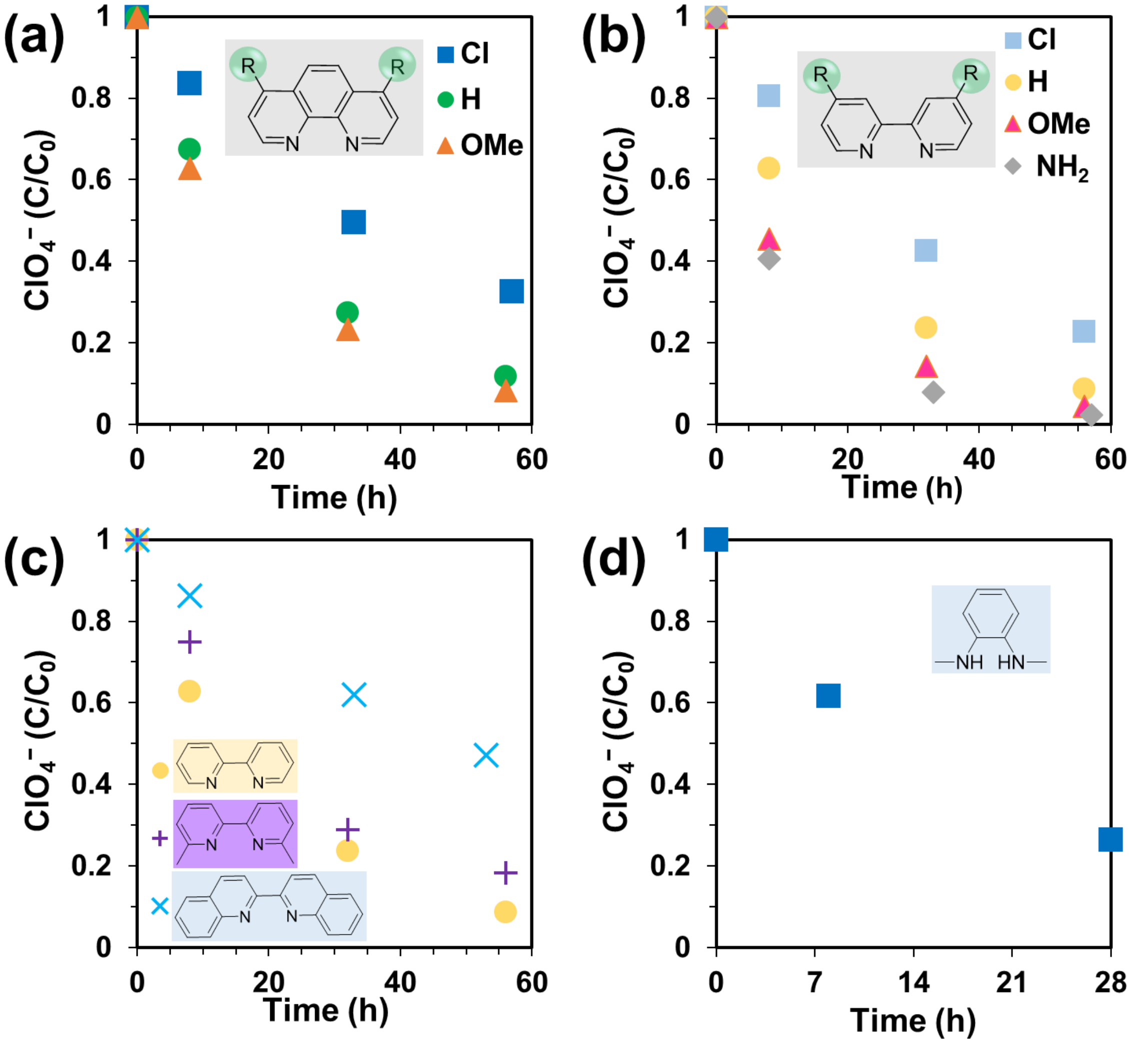

Figure S1. Time profiles for aqueous ClO_4^- reduction by Ru catalysts with aromatic ligands added. Reaction conditions: 2 g L^{-1} of a commercial 5 wt % Ru/C with 1 mM ligand added in the suspension, 1 mM ClO_4^-, pH 7, 1 atm of H_2, 20 °C.

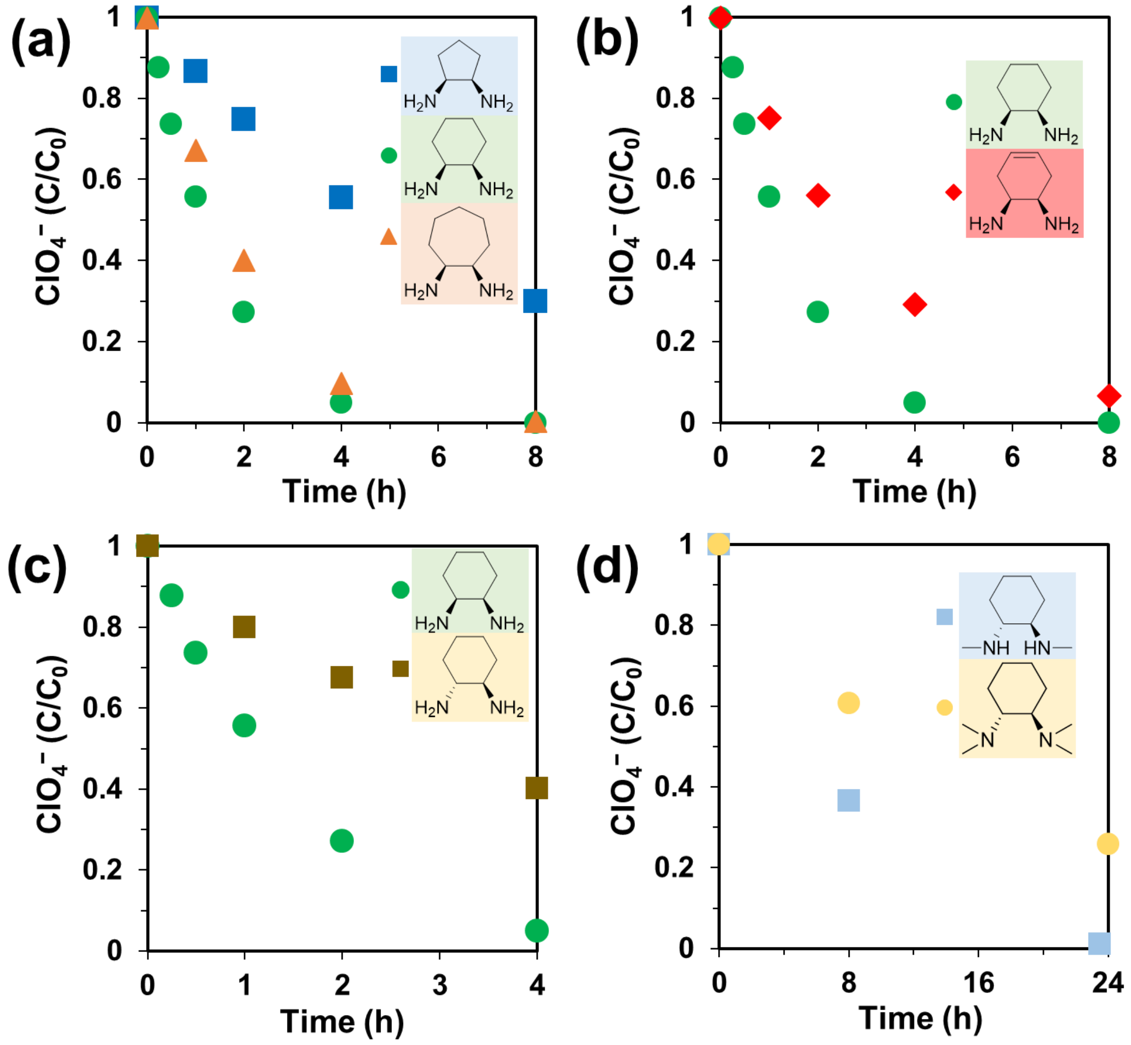

Figure S2. Time profiles for aqueous ClO_4^- reduction by Ru catalysts with diaminocycloalkanes (and a diaminocycloalkene) added. Reaction conditions: 2 g L^{-1} of a commercial 5 wt % Ru/C with 1 mM ligand added in the suspension, 1 mM ClO_4^-, pH 7, 1 atm of H_2, 20 °C.

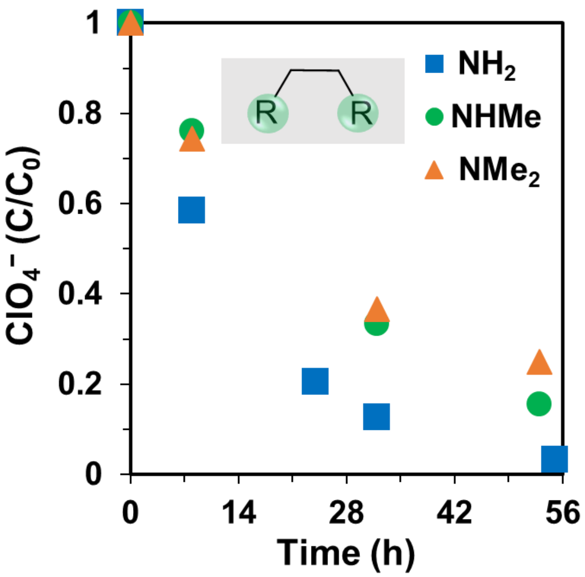

Figure S3. Time profiles for aqueous ClO_4^- reduction by Ru catalysts with linear aliphatic diamines added. Reaction conditions: 2 g L^{-1} of a commercial 5 wt % Ru/C with 1 mM ligand added in the suspension, 1 mM ClO_4^-, pH 7, 1 atm of H_2, 20 °C.

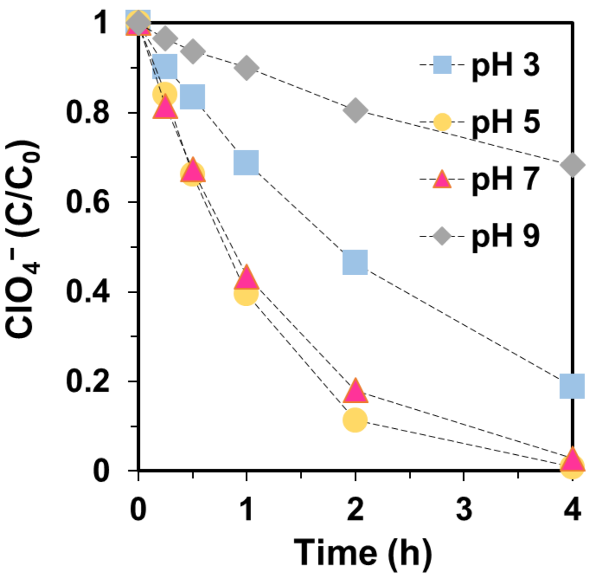

Figure S4. The effect of solution pH on the activity of [*cis*-DACH]Ru/C (no surface nitrogen functional groups). Reaction conditions: 2 g L^{-1} of the lab-prepared 5 wt % Ru/C, with 2 mM *cis*-DACH added in the suspension, 1 mM ClO_4^-, pH 7, 1 atm of H_2, 20 °C.

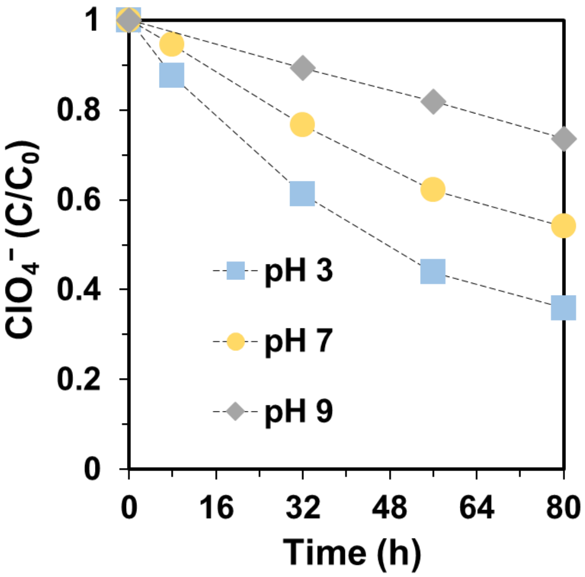

Figure S5. The effect of solution pH on the activity of a commercial Ru/C (no surface nitrogen functional groups; no *cis*-DACH added). Reaction conditions: 2 g L^{-1} of 5 wt % Ru/C, 1 mM ClO_4^-, 1 atm of H_2, 20 °C.

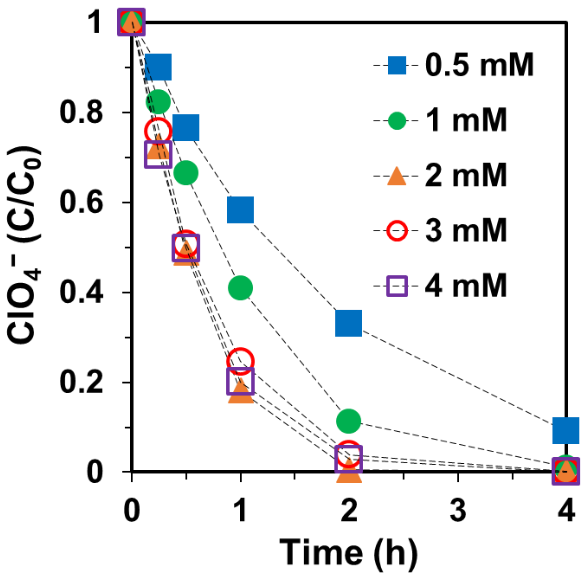

Figure S6. The effect of *cis*-DACH concentration. Reaction conditions: 2 g L^{-1} of 5 wt % Ru/NC, 1 mM ClO_4^-, pH 7, 1 atm of H_2, 20 °C.

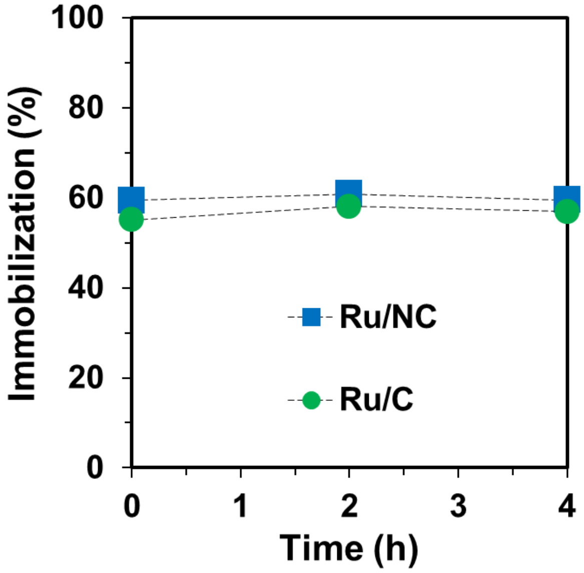

Figure S7. The ratio of the immobilized *cis*-DACH during ClO_4^- reduction. Reaction conditions: 2 g L^{-1} of 5 wt % Ru/C or Ru/NC with 2 mM *cis*-DACH added in the suspension, 1 mM ClO_4^-, pH 7, 1 atm of H_2, 20 °C.

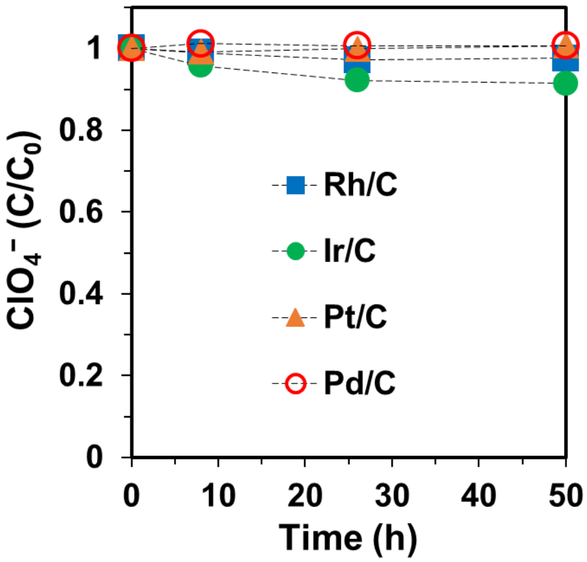

Figure S8. The reduction of ClO_4^- by other carbon-supported platinum group metal catalysts (details in **Table S5**). Reaction conditions: 2 g L^{-1} of individual catalyst with 2 mM *cis*-DACH added in the suspension, 1 mM ClO_4^-, pH 7, 1 atm of H_2, 20 °C.

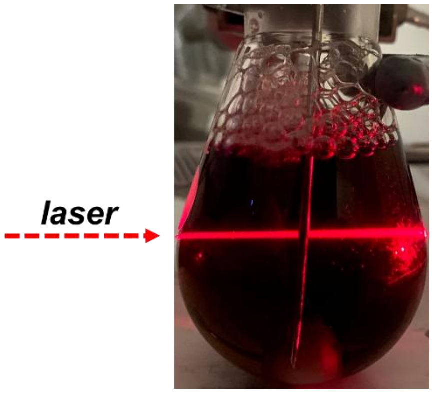

Figure S9. Tyndall effect by Ru colloids (1 mM $RuCl_3$ reduced with 10 mM $NaBH_4$ in the presence of 10 mM *cis*-DACH).

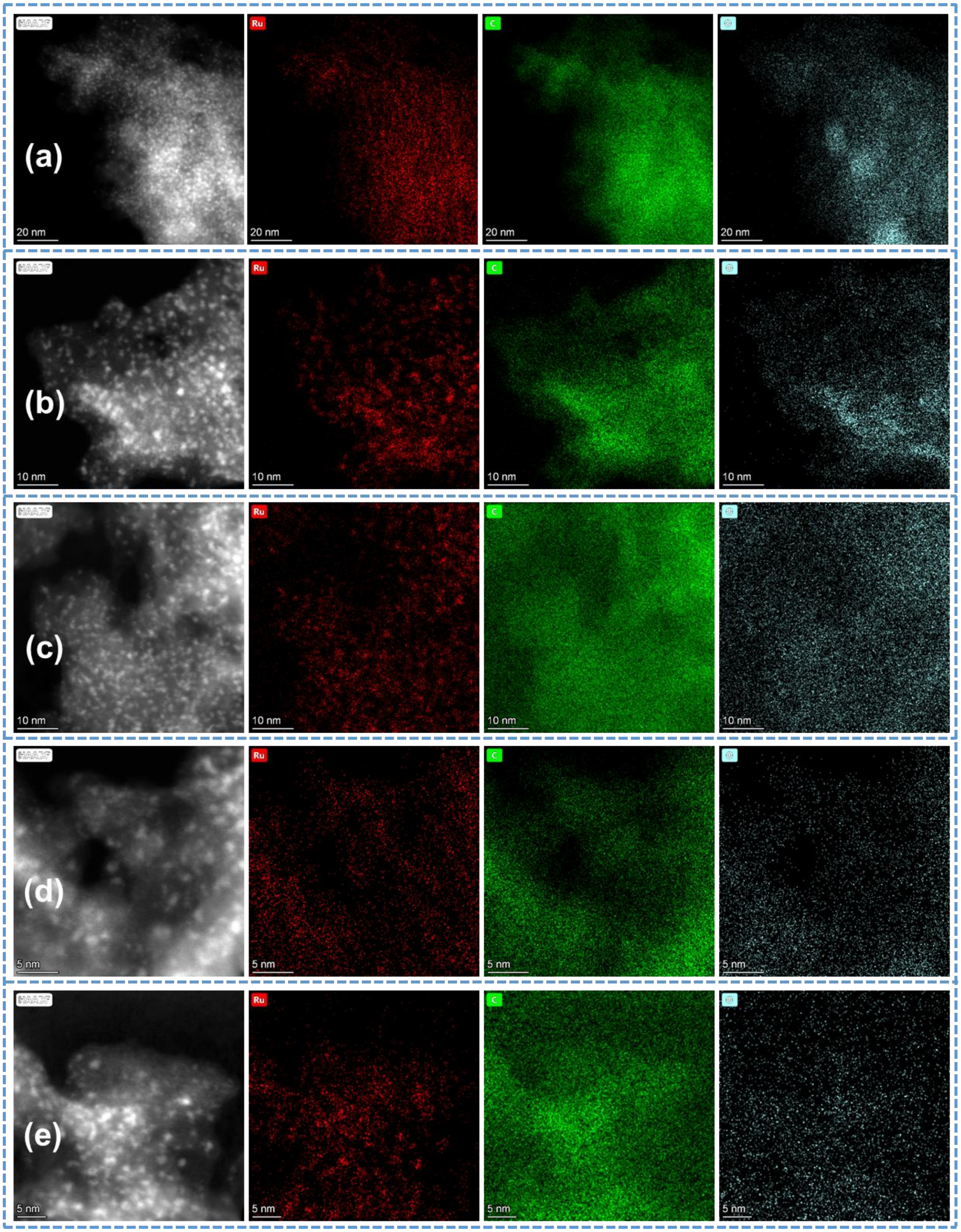

Figure S10. HAADF-STEM imaging and EDX mapping of the lab-prepared Ru/C.

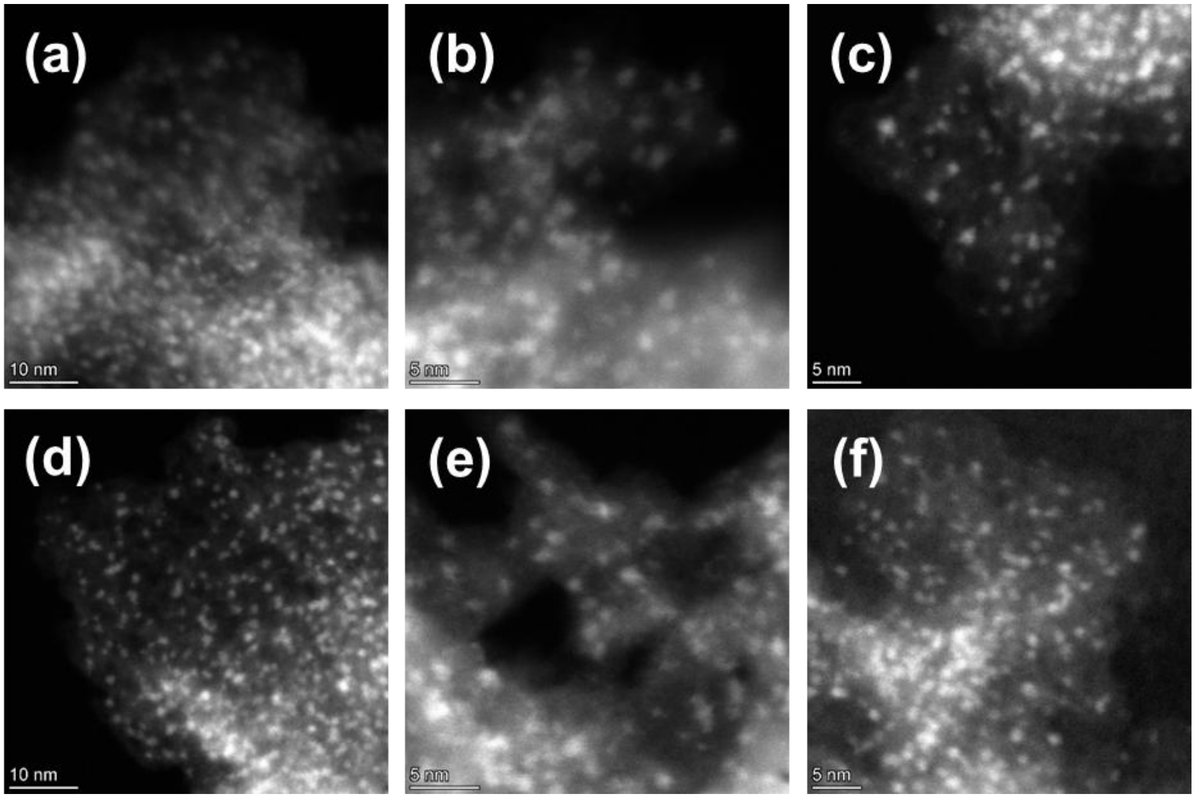

Figure S11. More HAADF-STEM imaging of the lab-prepared Ru/C.

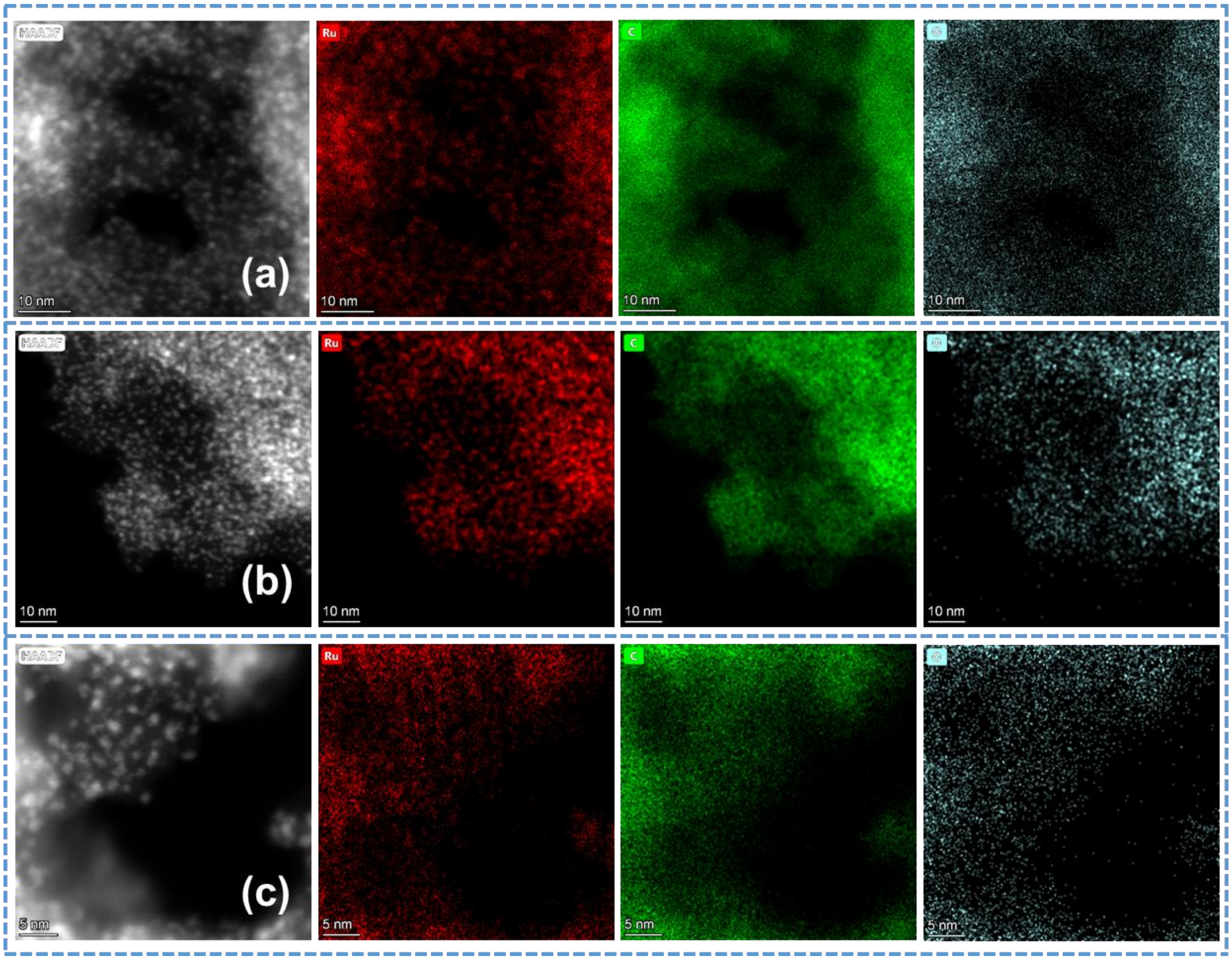

Figure S12. HAADF-STEM imaging and EDX mapping of Ru/NC. Note: Mapping nitrogen is challenging due to the strong absorption of the nitrogen K peak by carbon.

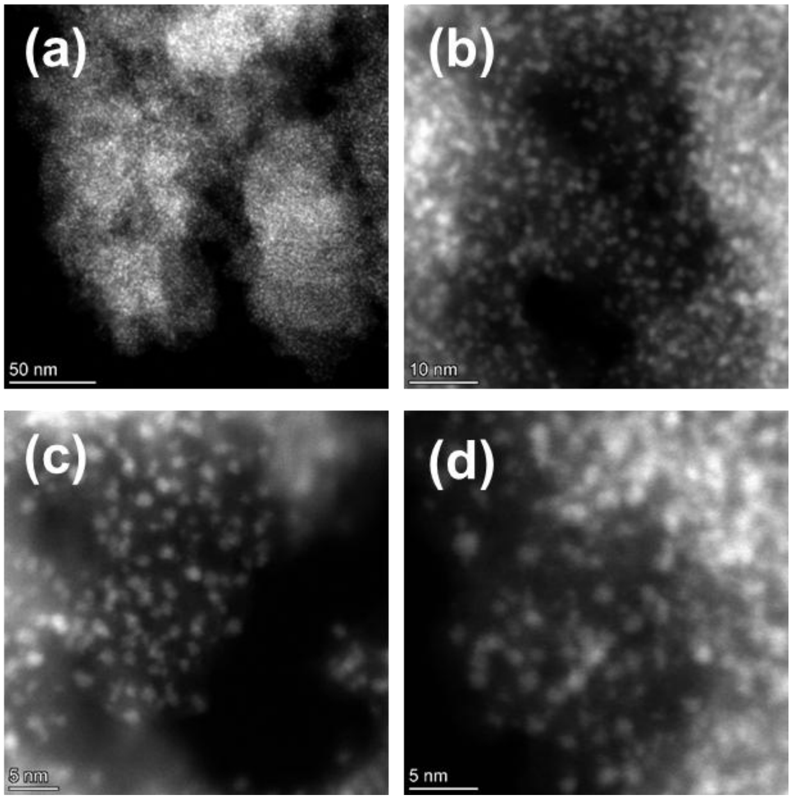

Figure S13. More HAADF-STEM imaging of Ru/NC.

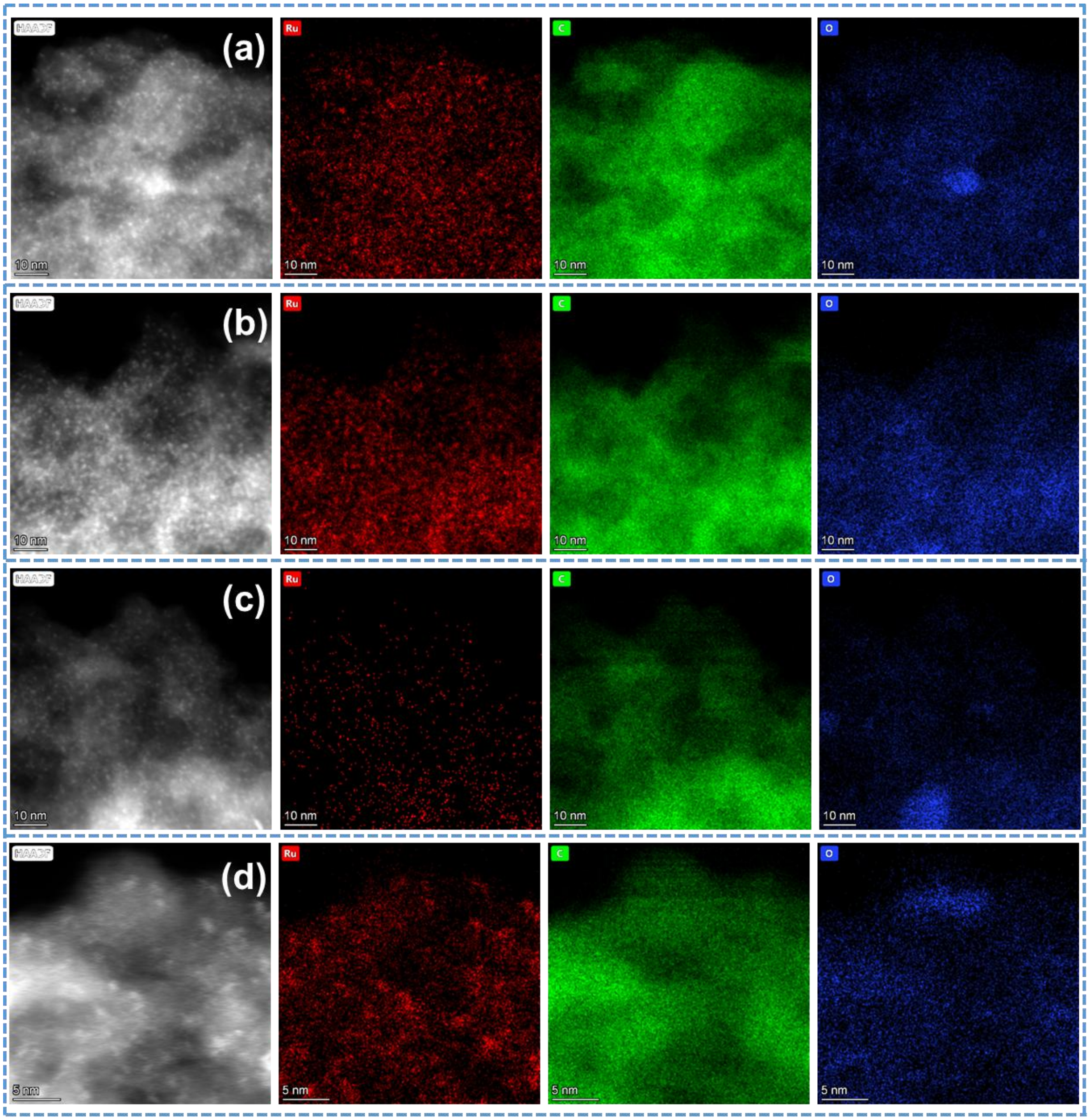

Figure S14. HAADF-STEM imaging and EDX mapping of [*cis*-DACH]Ru/C. Note: Mapping nitrogen is challenging due to the strong absorption of the nitrogen K peak by carbon.

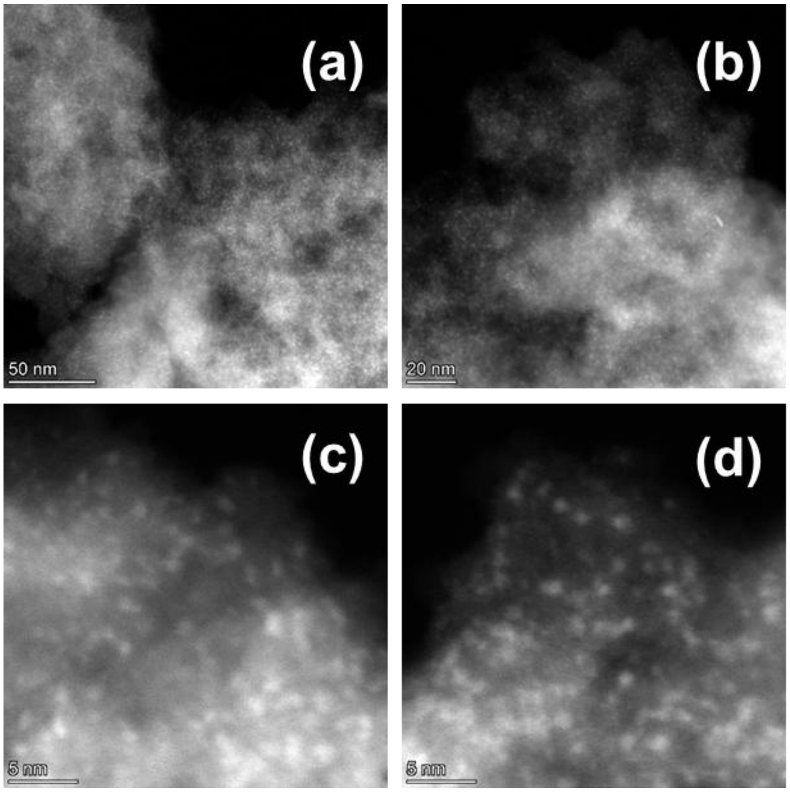

Figure S15. More HAADF-STEM imaging of [*cis*-DACH]Ru/C.

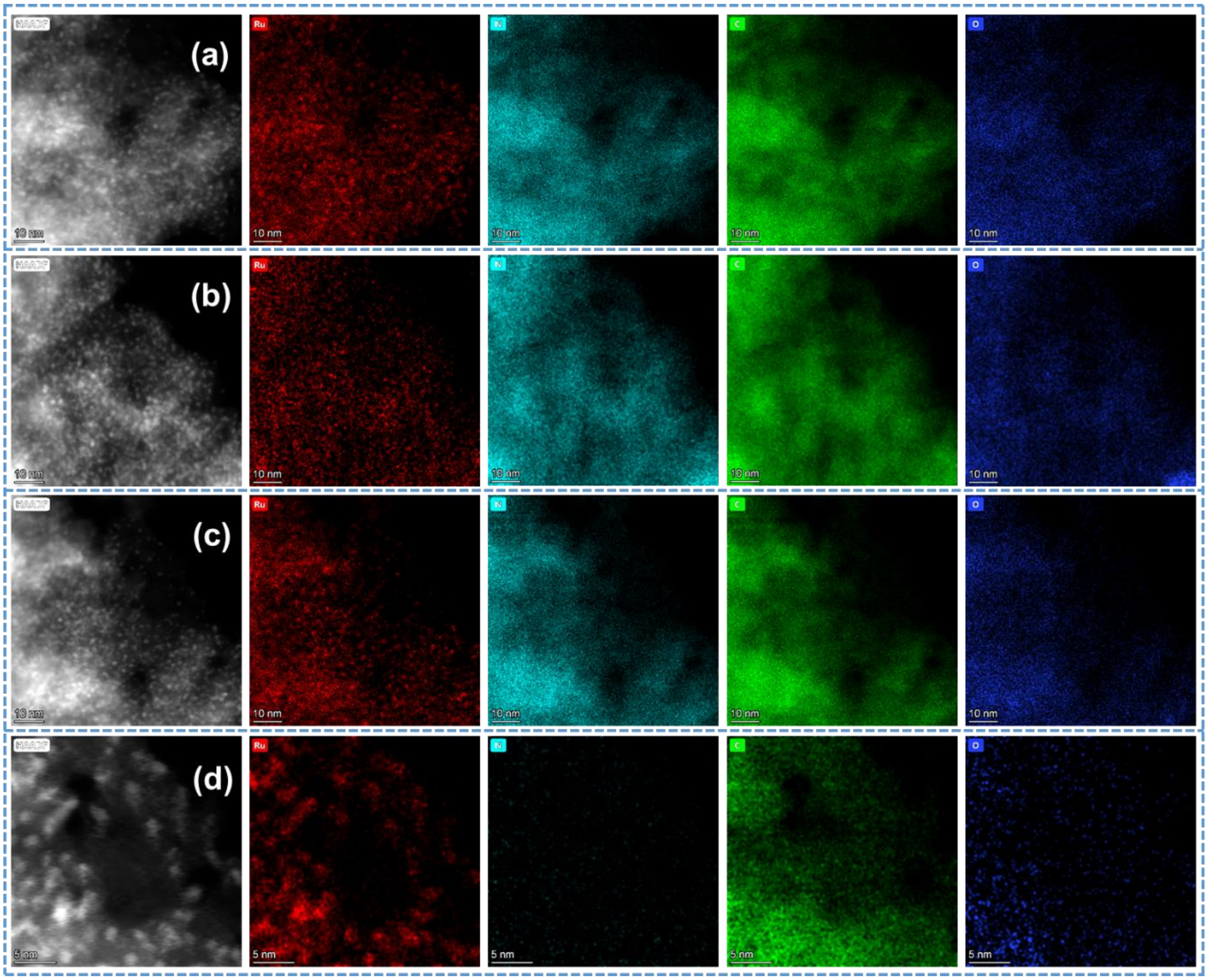

Figure S16. HAADF-STEM imaging and EDX mapping of [*cis*-DACH]Ru/NC. Note: The successful nitrogen mapping, in this case, is likely due to its high abundance, which overcame the challenge of the strong absorption of the nitrogen K peak by carbon.

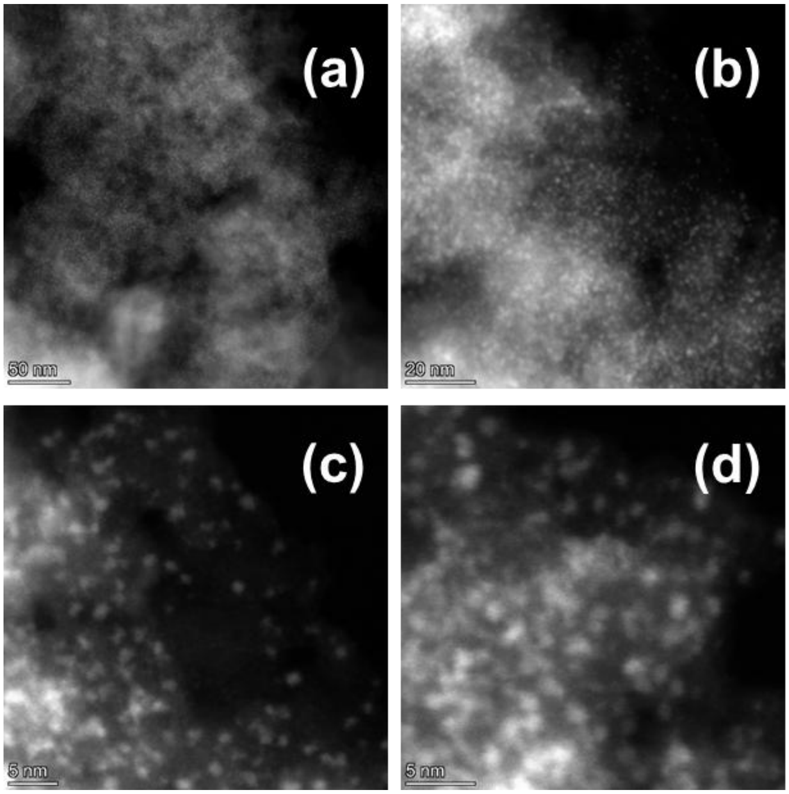

Figure S17. More HAADF-STEM imaging of [*cis*-DACH]Ru/NC.

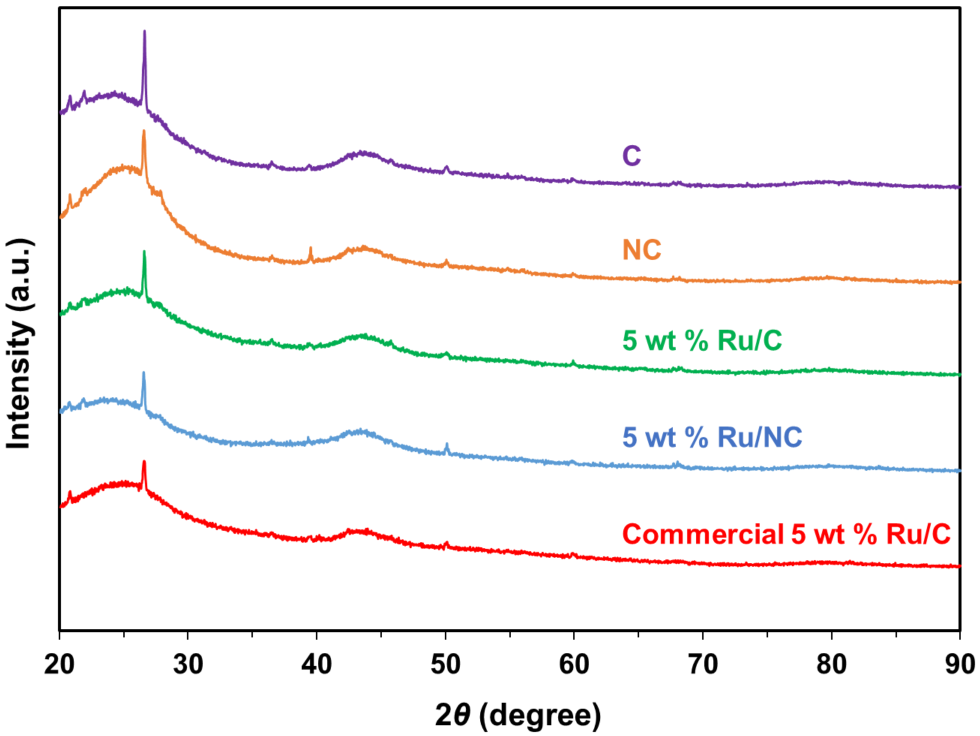

Figure S18. XRD patterns of catalyst supports and Ru catalysts.

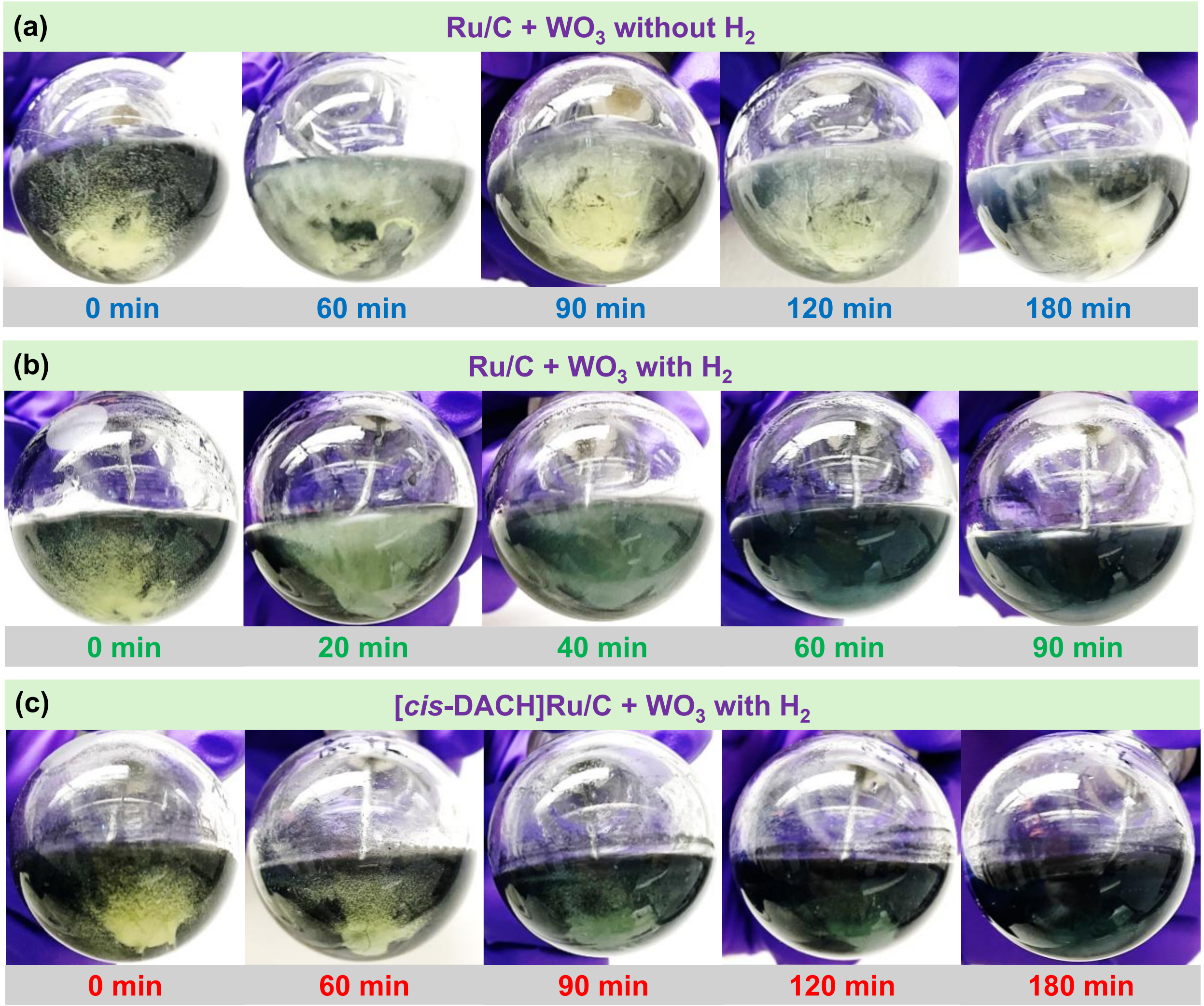

Figure S19. WO_3 color changes with (a) Ru/C without H_2, (b) Ru/C with H_2, and (c) [*cis*-DACH]Ru/C with H_2. Reaction conditions: 2 g L^{-1} of catalyst (5 wt% Ru), WO_3 powder (4 g L^{-1}), 2 mM of *cis*-DACH (if used), pH 7.0, 1 atm of H_2, 60 °C.

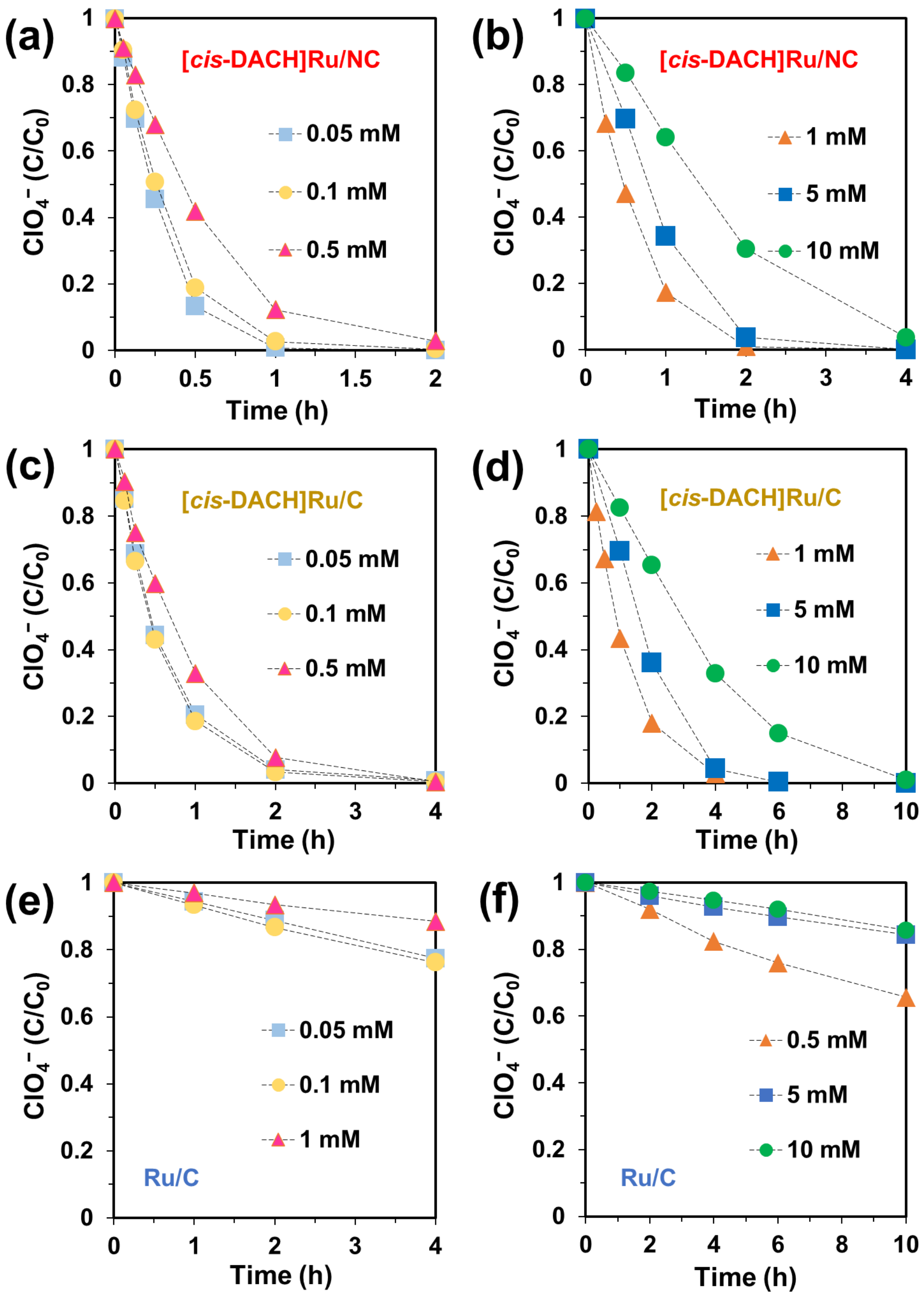

Figure S20. Catalytic reduction of ClO_4^- in various initial concentrations by (a+b) [*cis*-DACH]Ru/NC, (c+d) [*cis*-DACH]Ru/C, and (e+f) Ru/C. The initial rate of ClO_4^- reduction at various initial ClO_4^- concentrations were used for the Langmuir–Hinshelwood (LH) model fits (see **Text S2** for details). Reaction conditions: 2 g L^{-1} of 5 wt % Ru/NC or Ru/C with 2 mM of *cis*-DACH added in the suspension, pH 7, 1 atm of H_2, 20 °C.

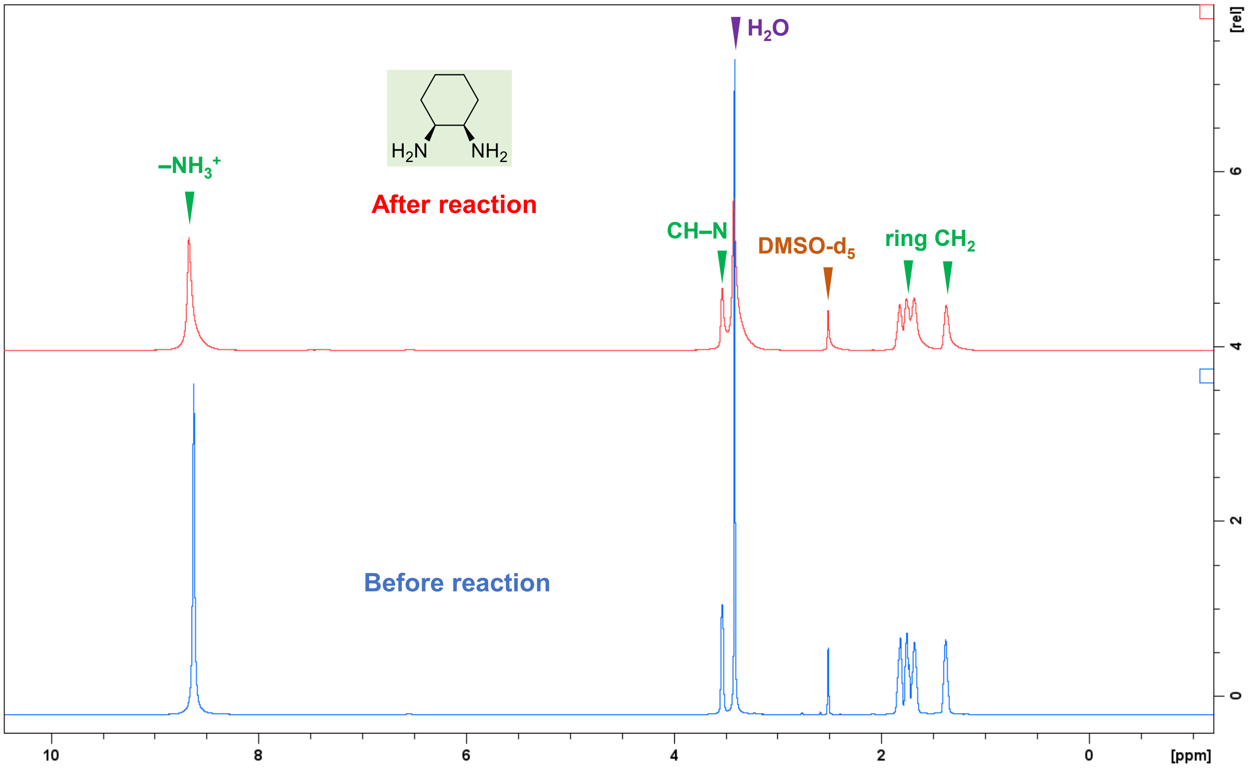

Figure S21. ^{1}H NMR spectra (600 MHz, DMSO-d^6) of re-collected *cis*-DACH (a) after and (b) before the catalytic reaction. Reaction Conditions: 20 mL of water, 2.0 g L^{-1} of 5 wt% Ru/C (Alfa Aesar #44338), 5 mM *cis*-DACH, 1 mM ClO_4^-, pH 7.0, 1 atm of H_2, 20 °C.

NMR sample preparation: after perchlorate reduction, the catalyst was separated from the water via vacuum filtration with analytical filter paper, and the filter cake was rinsed with three times of 10 mL DI water. The original filtrate (20 mL) and rinsates (10 mL × 3) were combined and acidified with 20 mM of HCl (to protonate the *cis*-DACH and avoid evaporation loss during the drying process). The water solution was dried by rotary evaporation (water batch at 60 °C), and the resulting solid was dissolved in DMSO-d^6 for NMR analysis. A "before reaction" control was prepared by adding HCl to the ligand stock solution at the same concentrations in the absence of Ru/C catalyst, followed by the same evaporation and dissolution procedures.

Conclusion: The ^{1}H NMR spectra of the recovered sample (after perchlorate reduction using Ru/C) showed the same resonances as the fresh ligand (processed in the control sample) and no new species, confirming that the *cis*-DACH ligand did not undergo any chemical transformation during the catalytic perchlorate reduction.

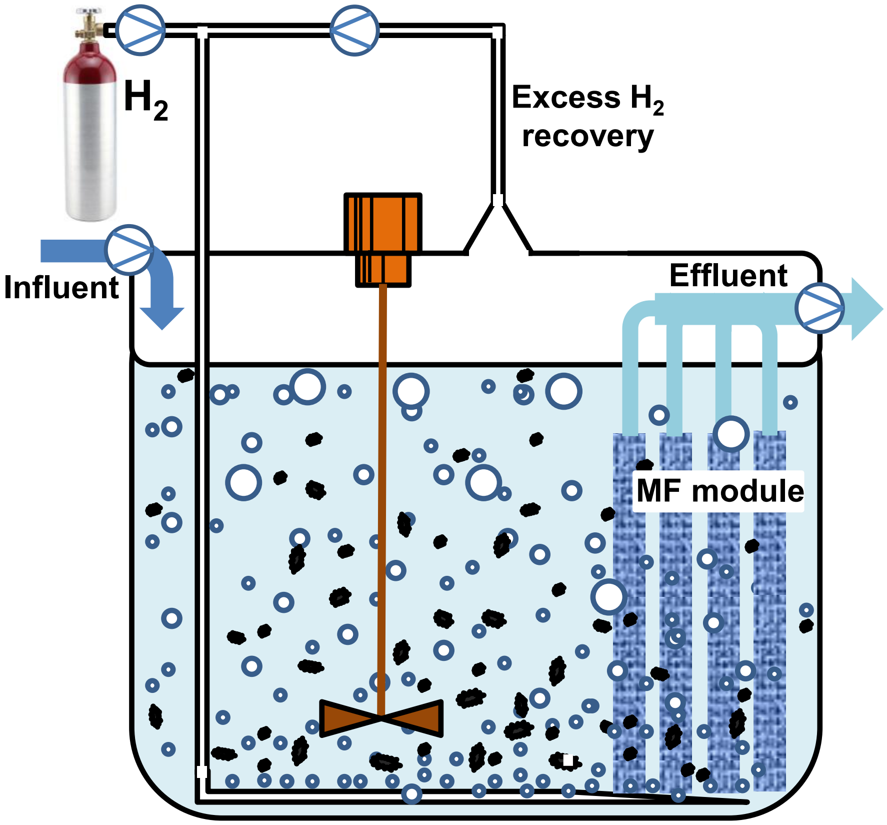

Figure S22. A proposed stirred-tank reactor design for catalytic perchlorate reduction. The reactor employs mechanical stirring to maintain a uniform suspension of catalyst (black particles) in water, with H_2 bubbles (open circles) continuously supplied through a gas diffuser. A submerged microfiltration (MF) module retains the solid catalyst within the reactor while withdrawing treated effluent.

Text S1−S3 Referred in the Main Text

Text S1. TON and TOF Calculation

The calculation assumed that the Ru sites reacted with all ClO_x^- (x = 4, 3, 2, and 1) substrates. The turnover number (TON) was calculated as

$$TON = 4 \times [ClO_4^-]_0 \times M_w / (L_{cat.} \times C_{metal} \times D_{metal})$$

where $[ClO_4^-]_0$ is the initial concentration of perchlorate (mol L^{-1}), M_w is the atomic mass of Ru (g mol^{-1}), $L_{cat.}$ is the loading of catalyst powder (g L^{-1}), C_{metal} is the metal content, and D_{metal} is the metal dispersion.

The initial turnover frequency (TOF_0, in h^{-1}) was calculated as

$$TOF_0 = 4 \times ([ClO_4^-]_0 - [ClO_4^-]_t) \times M_w / (L_{cat.} \times C_{metal} \times D_{metal} \times t)$$

where $[ClO_4^-]_t$ is the concentration at the first sampling point of reaction time t (h).

The TOF values reported in Table 1 were calculated based on metal dispersion determined by CO chemisorption on the ligand-free catalyst, and therefore do not account for Ru surface sites occupied by ligands. Consequently, the metal dispersion is likely overestimated, and the true TOF values are correspondingly higher than reported.

Text S2. Mass Transfer Analysis and Langmuir-Hinshelwood Kinetics Modeling

The LH kinetic model assumes that the surface reaction is the rate-limiting step of the overall reaction. This implies that the transfer of ClO_4^- from the bulk solution to the liquid-solid interface occurs rapidly compared to the chemical reactions at the surface. It is thus necessary to evaluate internal and external mass transfer to confirm this assumption.

Internal Mass Transfer

To evaluate the impact of pore diffusion resistance on measured reaction rates, we used the Weisz-Prater (WP) criterion.[14, 15] In general, the WP criterion and WP parameter (C_{WP}) are described as follows:

If $C_{WP} = \frac{k_{obs}\tau R^2}{D\theta} < 1$, the internal mass transfer limitation is negligible;

If $C_{WP} = \frac{k_{obs}\tau R^2}{D\theta} > 1$, the internal mass transfer limitation is significant.

where k_{obs} is the observed pseudo-first-order rate constant of heterogeneous reaction (min^{-1}), τ is the tortuosity factor of the catalyst particle, R is the radius of the catalyst particle (m), D is the diffusion coefficient of reactant in bulk solution (m^2 s^{-1}), and θ is the porosity of the catalyst particle.

To evaluate C_{WP} conservatively, we used the highest reaction rate (i.e., 3.175 h^{-1}) measured in this study. The typical range for parameters τ and θ are 2−10 and 0.2−0.7, respectively. For the conservative calculation, we used 10 and 0.2 as the estimated values for τ and θ, respectively.[16, 17] The catalyst particle diameter is 25 μm (provided by the vendor of carbon support). The diffusion coefficient of ClO_4^- in bulk solution is 1.79×10^{-9} m^2 s^{-1}.[18] Hence, C_{WP} was calculated as:

$$C_{WP} = \frac{k_{obs}\tau R^2}{D\theta} = \frac{3.175\ h^{-1}\times10\times(\frac{25}{2}\times10^{-6}\ m)^2}{1.79\times10^{-9}\ m^2\ s^{-1}\times3600\ s\ h^{-1}\times0.2} = 0.0038\ "1 \quad (1)$$

Therefore, the internal mass transfer limitation is negligible.

External Mass Transfer

To estimate the mass transfer coefficient ($k_{aq/s}$) of ClO_4^- between the catalyst surface and the aqueous solution, we first calculated the Sherwood number (Sh).[19, 20]

$$Sh = \left[2 + 0.4\left(\frac{\varepsilon d_p^4}{v^3}\right)^{0.25} Sc^{0.33}\right] \cdot \phi_c \tag{2}$$

In **Eq. 2**, ε is the rate of flow energy supply per unit mass of liquid ($m^2\ s^{-3}$), dp is the diameter of the catalyst particle (m), v is the kinematic viscosity of water ($1.003 \times 10^{-6}\ m^2\ s^{-1}$ at 20°C), Sc is Schmidt number, and ϕ_c is Carman's surface factor (assume the catalyst particle is spherical, ϕ_c = 1). We evaluated Sc and ε using the following equations:

$$Sc = \frac{v}{D} = \frac{1.003 \times 10^{-6}\ m^2\ s^{-1}}{1.79 \times 10^{-9}\ m^2\ s^{-1}} = 560.34 \tag{3}$$

$$\varepsilon = \frac{N_p \cdot l^5 \cdot n^3}{V} = \frac{5 \times (3\ cm)^5 \times (27\ s^{-1})^3}{50\ cm^3} = 0.525\ m^2\ s^{-3} \tag{4}$$

where N_p is the power number (normally $Np \approx 5.0$)[21] , l is the length of the stir bar ($l \approx 3\ cm$), n is the rotating speed of the stir bar ($n = 360\ round\ min^{-1} = 6\ round\ s^{-1}$), and V is the volume of the reactor ($V = 50\ cm^3$).

The Sherwood number was then calculated using the calculated values of Sc and ε:

$$Sh = \left[2 + 0.4\left(\frac{0.525\ m^2\ s^{-3} \times (25 \times 10^{-6}\ m)^4}{(1.003 \times 10^{-6}\ m^2\ s^{-1})^3}\right)^{0.25} 560.34^{0.33}\right] \cdot 1 = 4.168 \tag{5}$$

And $k_{aq/s}$ was calculated by **Eq. 6**:

$$k_{aq/s} = \frac{D}{d_p} \cdot Sh = \frac{1.79 \times 10^{-9}\ m^2\ s^{-1}}{25 \times 10^{-6}\ m} \cdot 4.168 = 2.98 \times 10^{-4}\ m\ s^{-1} \tag{6}$$

The geometric surface area of the catalyst per volume of solution (a)[22] is calculated by using **Eq.7**:

$$a = \frac{SA_p \times M}{\rho_p \times V_p} \times \frac{1}{V_R} = \frac{4\pi \times (12.5 \times 10^{-6}\ m)^2 \times 0.1\ g}{2 \times 10^6\ g\ m^{-3} \times \frac{4\pi}{3}(12.5 \times 10^{-6}\ m)^3} \times \frac{1}{50 \times 10^{-6}\ m^3} = 240\ m^{-1} \tag{7}$$

where SA_p is the geometric surface area of one catalyst particle (m^2), M is the mass of catalyst in the reduction test (0.1 g), ρ_p is the density of the catalyst ($g\ m^{-3}$), V_p is the volume of one catalyst particle (m^3), and V_R is the volume of the reactor (50 mL).

For the mass transfer rate calculation, the geometric surface area of the catalyst per volume of the solution was multiplied by the mass transfer coefficient.

$$k_{aq/s} \cdot a = 2.98 \times 10^{-4}\ m\ s^{-1} \cdot 240\ m^{-1} = 0.07152\ s^{-1} = 257.472\ h^{-1} \tag{8}$$

Based on the estimated values, the external mass transfer rate is significantly higher than the observed rate constant $k_{obs} = 3.175\ h^{-1}$ for ClO_4^- reduction. As a result, the impact of external mass transfer on the reaction rates can be considered negligible.

Langmuir-Hinshelwood Model

The Langmuir-Hinshelwood (LH) model was used to analyze the kinetic data of Ru/C, [*cis*-DACH]Ru/C, and [*cis*-DACH]Ru/NC catalysts. The catalytic reduction of ClO_4^- involves multiple steps, including reversible adsorption of ClO_4^- onto the unoccupied active sites, reduction of ClO_4^- on active sites, and desorption of products. Assuming that (i) the surface reaction is the rate-limiting step, (ii) ClO_4^- and H_2 are adsorbed on the catalyst surface non-competitively, (iii) there is no activity loss of the Ru sites, and (iv) the adsorption of ClO_4^- on activated carbon is minimal, the surface reaction rate (r) can be simplified to a single site model, and the global reaction rate can be defined as:

$$r = k_2[AS] = k_2\theta[S] \quad (9)$$

where k_2 is the surface reaction rate constant, $[AS]$ and $[S]$ denote the surface concentration of occupied sites (ClO_4^- associated with metal active sites) and total active sites (occupied plus unoccupied sites), respectively. θ is the surface coverage, which is defined as $\theta = [AS]/[S]$. Since the adsorption and desorption of ClO_4^- are assumed to be fast, the steady-state approximation can be applied to AS:

$$\frac{d[AS]}{dt} = k_1[A][S](1-\theta) - k_{-1}[S](\theta) - k_2[S](\theta) = 0 \quad (10)$$

where k_1 and k_{-1} are the adsorption rate constant and desorption rate constant of ClO_4^-, respectively, and $[A]$ denotes the aqueous ClO_4^- concentration.

Hence, θ is solved as:

$$\theta = \frac{k_1[A]}{k_1[A]+k_{-1}+k_2} \quad (11)$$

Since the surface reaction is assumed to be the rate-limiting step (i.e., k_2 "$k_1[A]$ and k_2" k_{-1}), θ can be simplified to:

$$\theta = \frac{k_1[A]}{k_1[A]+k_{-1}} \quad (12)$$

From **Eq. 12** and **Eq. 9**, r can be expressed in terms of $[A]$:

$$r = \frac{K_1k_3[A]}{K_1[A]+1} \quad (13)$$

where $K_1 = k_1/k_{-1}$ is the adsorption equilibrium constant, $k_3 = k_2[S]$ is affected by the total amount of the Ru active sites.

We carried out ClO_4^- reduction experiments at pH 7 using varying initial concentrations of ClO_4^- (Figure S19). The initial rates of ClO_4^- reduction r (mmol g_{cat}^{-1} h^{-1}) were fit to **Eq. 13** (Figure 3g). The adsorption equilibrium constant K_1 (L $mmol^{-1}$) and rate constant k_3 (mmol g_{cat}^{-1} h^{-1}) for Ru/C, [*cis*-DACH]Ru/C, and [*cis*-DACH]Ru/NC catalysts were listed in Figure 3h.

Text S3. Catalyst Characterization

X-ray photoelectron spectroscopy (XPS): The dried catalyst powder was loaded onto a copper conductive tape inside the glove bag and transported with an anaerobic tube to the XPS instrument (AXIS Supra Kratos Analytical, equipped with monochromatized Al Kα source) at the UC Irvine Materials Research Institute. The sp^2 C 1s peak (284.5 eV) of the carbon support was used as the reference for binding energy calibration. CasaXPS software (version 2.3.19) was used for the fit of XPS spectra in the resolution of 0.1 eV.

Diffuse reflectance FT-IR spectroscopy (DRIFTS): In situ DRIFTS experiments of CO adsorption-desorption on Ru/Al_2O_3 and [*cis*-DACH]Ru/Al_2O_3 were performed on a Nicolet iS50 FTIR spectrometer with a liquid nitrogen-cooling MCT detector. The backgrounds and spectra taken had a resolution of 4 cm^{-1}. Each spectrum was the average of 100 scans, with the background subtracted automatically. Before the measurement, 30 mg of the powder sample was loaded into a high-temperature IR cell (PIKE DiffusIR cell with ZnSe windows) and pretreated in an Ar flow of 30 mL min^{-1} at 200 °C for 1 h. Subsequently, the sample was controlled at 25 °C and purged by Ar (50 mL min^{-1}) for 30 min before collecting the background spectrum. For CO adsorption at 25 °C, the feed stream of 1% CO balanced with Ar was introduced into the cell with a flow rate of 50 mL min^{-1} for 30 min to achieve the saturated CO adsorption. The CO flow was then discontinued, while Ar (50 mL min^{-1}) was kept flowing for 15 min to remove the gaseous and weakly adsorbed CO.

Scanning transmission electron microscope (STEM): Before being characterized by STEM (FEI Titan Themis 300 equipped with an energy dispersive X-ray spectrometer (EDX) system), the catalyst powder was resuspended and sonicated in distilled water to further reduce the size. The STEM images were acquired with a high-angle annular dark-field (HAADF) detector. Nano Measurer software package was used for the statistical analysis of average particle size in the STEM images.

Supplemental analyses: Elemental analyses of Ru and N were conducted by the Microanalysis Laboratory, University of Illinois at Urbana-Champaign. After microwave digestion, the Ru content in the catalyst powder was measured by inductively coupled plasma–optical emission spectrometry (ICP–OES, PerkinElmer Optima 8300). The N content was determined by an elemental analyzer (Exeter Analytical, Inc., Model CE440). The specific surface area of Ru particles in the catalysts were measured by CO pulse titration experiments on a Quantachrome Autosorb-iQ physisorption-chemisorption instrument with the surface Ru:CO stoichiometry of 12:7.[13] X-ray diffraction (XRD) characterization was performed with a Panalytical Empyrean instrument (45 kV/40 mA) equipped with a Cu Kα source.

References

(1) Yoshida, D.; Liu, J.; Huang, K.; Otomo, R.; Kamiya, Y. Reduction of perchlorate in neutral water over a ceria-supported ruthenium catalyst towards the purification of contaminated water. *Appl. Catal. A: Gen.* **2023,** *649*, 118982.
(2) Liu, J.; Choe, J. K.; Wang, Y.; Shapley, J. R.; Werth, C. J.; Strathmann, T. J. Bioinspired complex-nanoparticle hybrid catalyst system for aqueous perchlorate reduction: Rhenium speciation and its influence on catalyst activity. *ACS Catal.* **2015,** *5* (2), 511-522.
(3) Ren, C.; Yang, P.; Sun, J.; Bi, E. Y.; Gao, J.; Palmer, J.; Zhu, M.; Wu, Y.; Liu, J. A bioinspired molybdenum catalyst for aqueous perchlorate reduction. *J. Am. Chem. Soc.* **2021,** *143* (21), 7891-7896.
(4) Hurley, K. D.; Shapley, J. R. Efficient heterogeneous catalytic reduction of perchlorate in water. *Environ. Sci. Technol.* **2007,** *41* (6), 2044-2049.
(5) Hurley, K. D.; Zhang, Y.; Shapley, J. R. Ligand-enhanced reduction of perchlorate in water with heterogeneous Re−Pd/C catalysts. *J. Am. Chem. Soc.* **2009,** *131* (40), 14172-14173.
(6) Crowell, W. R.; Yost, D. M.; Roberts, J. D. The catalytic effect of osmium compounds on the reduction of perchloric acid by hydrobromic acid. *J. Am. Chem. Soc.* **1940,** *62* (8), 2176-2178.
(7) Haight Jr, G. Mechanism of the tungstate catalyzed reduction of perchlorate by stannous chloride. *J. Am. Chem. Soc.* **1954,** *76* (18), 4718-4721.
(8) Abu-Omar, M. M.; Espenson, J. H. Facile abstraction of successive oxygen atoms from perchlorate ions by methylrhenium dioxide. *Inorg. Chem.* **1995,** *34* (25), 6239-6240.
(9) Abu-Omar, M. M.; McPherson, L. D.; Arias, J.; Béreau, V. M. Clean and efficient catalytic reduction of perchlorate. *Angew. Chem., Int. Ed.* **2000,** *112* (23), 4480-4483.
(10) Cao, J.; Elliott, D.; Zhang, W.-x. Perchlorate reduction by nanoscale iron particles. *J. Nanopart. Res.* **2005,** *7*, 499-506.
(11) Gu, B.; Dong, W.; Brown, G. M.; Cole, D. R. Complete degradation of perchlorate in ferric chloride and hydrochloric acid under controlled temperature and pressure. *Environ. Sci. Technol.* **2003,** *37* (10), 2291-2295.
(12) Haight Jr, G.; Sager, W. Evidence for preferential one-step divalent changes in the molybdate-catalyzed reduction of perchlorate by stannous ion in sulfuric acid solution. *J. Am. Chem. Soc.* **1952,** *74* (23), 6056-6059.
(13) Chen, Q.; Liu, J.; Zhou, X.; Shang, J.; Zhang, Y.; Shao, X.; Wang, Y.; Li, J.; Chen, W.; Xu, G.; Wu, K. Unveiling structural evolution of co adsorption on Ru(0001) with high-resolution stm. *J. Phys. Chem. C.* **2015,** *119* (16), 8626-8633.
(14) Shuai, D.; Choe, J. K.; Shapley, J. R.; Werth, C. J. Enhanced activity and selectivity of carbon nanofiber supported pd catalysts for nitrite reduction. *Environmental science & technology* **2012,** *46* (5), 2847-2855.
(15) Weisz, P.; Prater, C. Interpretation of measurements in experimental catalysis. *J Adv. Catal* **1954,** *6* (143), 60390-9.
(16) Davie, M. G.; Reinhard, M.; Shapley, J. R. Metal-catalyzed reduction of *N*-nitrosodimethylamine with hydrogen in water. *Environ. Sci. Technol.* **2006,** *40* (23), 7329-7335.
(17) Satterfield, C. Mass Transport in Heterogeneous Catalysis; MIT Press: Cambridge, MA, 1970.
(18) Heil, S. R.; Holz, M.; Kastner, T. M.; Weingärtner, H. Self-diffusion of the perchlorate ion in aqueous electrolyte solutions measured by ^{35}Cl NMR spin–echo experiments. *J. Chem. Soc., Faraday Trans.* **1995,** *91* (12), 1877-1880.

(19) Sänger, P.; Deckwer, W. D. Liquid—solid mass transfer in aerated suspensions. *Chem. Eng. J.* **1981,** *22* (3), 179-186.
(20) Sano, Y.; Yamaguchi, N.; Adachi, T. Mass transfer coefficients for suspended particles in agitated vessels and bubble columns. *J. Chem. Eng. Jpn.* **1974,** *7* (4), 255-261.
(21) Bates, R. L.; Fondy, P. L.; Corpstein, R. R. Examination of some geometric parameters of impeller power. *Ind. Eng. Chem. Proc. Des. Dev.* **1963,** *2* (4), 310-314.
(22) Liu, B.; Yao, H.; Song, W.; Jin, L.; Mosa, I. M.; Rusling, J. F.; Suib, S. L.; He, J. Ligand-free noble metal nanocluster catalysts on carbon supports via "soft" nitriding. *J. Am. Chem. Soc.* **2016,** *138* (14), 4718-4721.
